# Neurobiology of Pet Loss Grief: A Review from a One Health Perspective

**DOI:** 10.3390/ani16193129

**Published:** 2026-10-06

**Authors:** Thanh Huu Phan Ngo, Jiwon Oh, Hyeong Jun Kim, Wan Lee

**Affiliations:** 1 Department of Biochemistry, Dongguk University College of Medicine, 123 Dongdae-ro, Gyeongju 38066, Republic of Korea; thngo139@gmail.com (T.H.P.N.); jiwon8757@naver.com (J.O.); bdbd147@naver.com (H.J.K.); 2 Section of Molecular and Cellular Medicine, Medical Institute of Dongguk University, Gyeongju 38066, Republic of Korea

**Keywords:** pet loss grief, companion animal bereavement, human–animal bond, disenfranchised grief, oxytocin, HPA axis, neuroinflammation, prolonged grief disorder, One Health, cultural adaptation

## Abstract

The loss of a companion animal can provoke grief whose intensity rivals human bereavement, yet its biological basis remains largely uncharted. This structured narrative review synthesizes neurobiological, endocrinological, immunological, and psychosocial evidence into a mechanistic framework offered not as established fact but as a source of testable hypotheses. We trace the substrates of the human–animal bond across six interacting systems and propose how disruption of each upon an animal’s death may drive grief. Three carry comparatively robust analogical support (oxytocinergic signaling, hypothalamic–pituitary–adrenal [HPA] axis regulation, and neuroinflammatory activation) and three are more exploratory (dopaminergic, serotonergic, and endocannabinoid pathways). Integrating extracted study-level data into transparent prioritization heuristics, we identify the HPA axis, assessed with its coupled oxytocinergic partner, as the most defensible first target for a direct biomarker study, a conclusion robust across three methodologically independent analyses. No direct pet-loss evidence yet supports any proposal, and this gap is the review’s central motivation. As applied context, we address euthanasia-related guilt, disenfranchised grief under East Asian norms (a tentative “double disenfranchisement” hypothesis that we put forward for testing), assessment instruments, evidence-graded therapies, priorities for developing Korean-language tools, and One Health research priorities.

## Simple Summary

For many people, a companion animal is a treasured family member, and its death can cause deep and lasting grief. Yet this grief is often dismissed by others as an overreaction, leaving bereaved owners feeling alone and unsupported. Surprisingly little is known about what actually happens in the body and brain when a beloved pet dies. This review brings together findings from two fields that have rarely been connected. One is research on how humans and animals form close bonds, and the other is research on how people grieve the loss of other humans. We describe how the same biological systems that build the bond with a pet, including hormones, stress responses, and reward pathways, may also drive the pain of losing one. We find that although these connections are biologically plausible, they have rarely been tested directly in grieving pet owners. We therefore identify priorities for future studies, including biological measures that could be tracked over time, and highlight how cultural attitudes may shape grief across countries. This work aims to help society recognize pet loss as genuine grief and to guide better support for those affected.

## 1. Introduction

When a companion animal dies, the owner’s world can come apart in ways that few anticipate, the owner included. Months of sadness, intrusive memories, and broken sleep are among the reactions that bereaved owners commonly describe. These reactions are neither rare nor confined to a sentimental minority. They are well documented and clinically relevant [1,2,3]. The literature on pet loss grief, though growing, is scattered across disciplines that have seldom looked at one another’s work, and it has almost nothing to say about bereaved owners in East Asian societies. The present review was undertaken to bridge that gap, and evidence that has until now remained dispersed across veterinary science, psychology, neuroscience, and public health is drawn together here.

The scale of companion animal ownership alone demands attention. In South Korea, the Animal and Plant Quarantine Agency under the Ministry of Agriculture, Food and Rural Affairs recorded roughly 3.29 million registered dogs and cats as of 2023, a figure that has risen each year [4]. Survey-based estimates of the total companion animal population run higher, but the registration data provide the most reliable lower bound. Worldwide, an estimated 57% of consumers reported owning a pet in the mid-2010s [5]. These animals are now commonly treated as family. The language of “fur baby” and “pet parent” is not mere affectation. It reflects a genuine psychological reorganization whose clinical implications medicine has been slow to absorb [6,7,8]. When such animals die, the grief response can mirror human bereavement in symptom profile and sometimes exceed it in severity [1,3]. A meaningful proportion of bereaved owners may satisfy DSM-5 criteria for persistent complex bereavement disorder [2], while depressive symptoms have been associated with heightened stress-induced inflammatory responses in humans [9]. This finding provides indirect context for the proposed pet loss mechanisms; it does not establish inflammatory or immune changes following pet loss. In a large UK study using quota-based sampling, 7.5% of pet-bereaved individuals met ICD-11 criteria for prolonged grief disorder, with the symptom structure showing full measurement invariance relative to human loss [10]. Taken together, these figures indicate that clinically significant distress occurs in a substantial minority of bereaved owners. The relationship also has deep roots. Dogs were the first species to be domesticated, and the many thousands of years since have involved coevolution rather than simple cohabitation, with consequences that are biological, behavioral, and social at once. Selection favored animals able to read human social signals and humans disposed to respond to them, so that the affiliative systems described in Section 3, which include the oxytocinergic, dopaminergic, and stress-regulatory circuits conserved across mammals, are engaged by a companion animal much as they are by a human companion. An attachment formed with an animal is therefore not a metaphorical version of a human attachment but a use of the same machinery, and that is the biological reason its rupture can produce a comparable response. This coevolutionary history is the theoretical foundation on which the rest of the review rests.

Yet this grief routinely goes unrecognized, and the obstacle to recognition is social rather than clinical. Doka’s [11] concept of disenfranchised grief, bereavement that society neither acknowledges nor sanctions, fits pet loss almost perfectly. In Korean clinical and social settings, the problem may run deeper. Cultural norms favoring emotional restraint create what we later describe as a potential double burden, in which owners grieve intensely but feel doubly prohibited from showing it [12,13]. Few healthcare professionals receive any training to recognize or respond to this particular form of loss [14].

**Figure 1:**
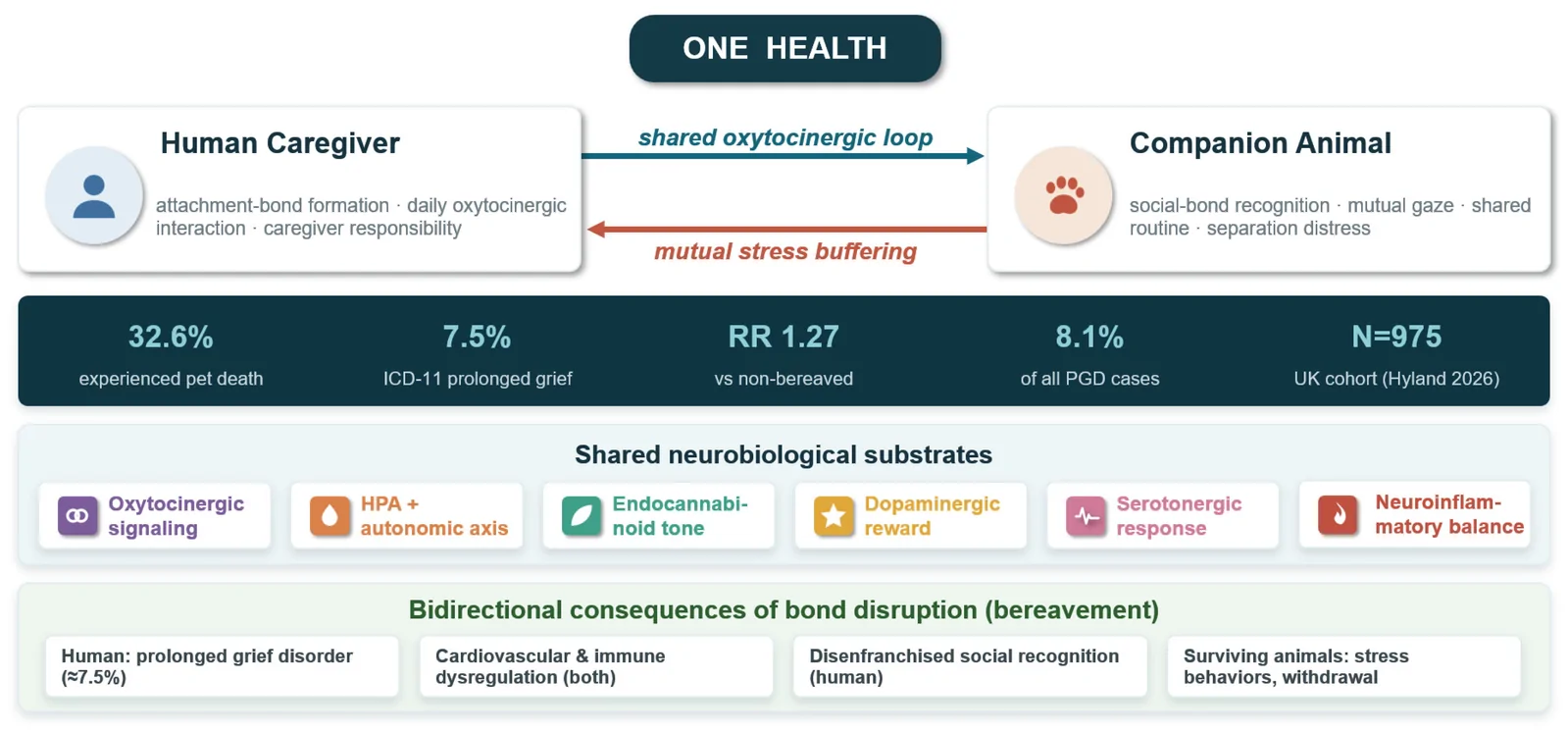
The One Health framework of companion-animal bereavement. Human caregivers and companion animals are joined through shared neurobiological substrates, oxytocinergic signaling, hypothalamic–pituitary–adrenal (HPA) + autonomic axis regulation, endocannabinoid tone, dopaminergic reward, serotonergic response, and neuroinflammatory balance. They are coupled through a shared oxytocinergic loop and mutual stress buffering, so that rupture of the attachment bond by death carries bidirectional consequences: prolonged grief with cardiovascular and immune dysregulation in humans, disenfranchised social recognition of the loss, and stress behaviors in surviving animals. Epidemiological anchors come from a UK quota sample [10] (N = 975), in which 32.6% had experienced the death of a pet, 7.5% met criteria for prolonged grief disorder, the relative risk of prolonged grief after pet loss was 1.27 versus non-bereaved, and pet loss accounted for 8.1% of all prolonged-grief cases. Each of the six neurobiological systems is assigned a color, and the same color is used for that system in every figure of this review. Data sources: the epidemiological values shown are taken from [10], and the neurobiological substrates summarized here are documented with citations in Section 3.

A substantial neurobiological gap sits at the center of these observations. We have extensive data on oxytocinergic and HPA-axis correlates of human social bonding. We know almost nothing about what happens biologically when someone loses an animal with whom they have shared daily oxytocin-mediated interactions for a decade or more. Closing this gap requires the kind of cross-disciplinary thinking that the One Health paradigm was designed to support (Figure 1) [15,16]. Veterinary medicine, psychology, neuroscience, and public health all need to be brought into the same analysis.

This review examines the neurobiological architecture of the human–animal bond and proposes how each system may become disrupted when that bond is severed by death. We also consider clinical phenomenology, epidemiology, and psychosocial context. We review assessment instruments and therapeutic approaches graded by evidence quality, discuss priorities for culturally adapted tool development in Korea, and discuss educational implications and future research directions.

Several prior reviews have addressed adjacent terrain, and two of them bear directly on the present one. Cleary and colleagues offered a systematic review focused on the clinical phenomenology of pet bereavement [17], and Packman and colleagues advanced the disenfranchised grief framework as it applies to animal loss [18]. Our contribution differs from these in both scope and integration, in that we synthesize neurobiological mechanisms across six interacting systems, extend the analysis to East Asian cultural contexts, and identify priorities for culturally adapted instrument development. To our knowledge, no prior review has combined these three elements—mechanistic framework, cultural extension, and instrument-development priorities—within a single One Health framing. It also differs in orientation from social-neuroscience reviews of human grief, which characterize bereavement-related brain circuitry in people but do not engage the companion-animal bond or the cross-species neurobiology that a One Health account requires. We regard this neurobiological framework and its data-driven prioritization as the primary contribution of the present review. The assessment instruments, therapeutic grading, cultural analysis, and educational recommendations that follow are developed as the applied context for which this framework is intended, rather than as independent aims.

## 2. Review Methodology

This structured narrative review follows the Scale for the Assessment of Narrative Review Articles (SANRA) quality criteria [19]. It is not a PRISMA-compliant systematic review, though we applied independent dual screening of titles and abstracts to reduce selection bias, as noted in the Limitations. A narrative synthesis is the appropriate design for this review because it aims to integrate mechanistically heterogeneous evidence, namely animal models, human bereavement studies, and human–animal-bond research, into a single hypothesis-generating framework, an objective that a PRISMA-style systematic review, which presumes a homogeneous body of studies with groupable outcomes, is not designed to serve.

Search strategy. We searched PubMed/MEDLINE, PsycINFO, Web of Science, Scopus, and Google Scholar from January 1980 to April 2026, without language restrictions. Search strings combined three conceptual blocks: (i) bereavement (“pet loss,” “companion animal bereavement,” “disenfranchised grief,” “complicated grief”); (ii) human–animal relationship (“human–animal bond,” “attachment to pet”); and (iii) neurobiology (“oxytocin,” “HPA axis,” “cortisol,” “dopamine,” “neuroinflammation,” “endocannabinoid”). For cultural-context searches, we added “Korea,” “Japan,” “China,” “Confucian,” and “East Asia” to the bereavement block.

Inclusion criteria. Original research, systematic reviews, meta-analyses, and narrative reviews addressing neurobiological, psychological, or cultural dimensions of pet loss grief or human–animal bonding were eligible. Book chapters were included where they represent seminal conceptualizations, for example, Doka’s disenfranchised grief framework [11].

Exclusion criteria. Conference abstracts without full text, editorial commentaries lacking original data, and articles dealing exclusively with animal welfare without reference to human bereavement were excluded.

Limitations of methodology. Titles and abstracts from database searches were independently screened by two reviewers (J.O. and H.J.K.) against the inclusion and exclusion criteria. Disagreements were resolved through discussion, and any case that remained unresolved was adjudicated by the corresponding author (W.L.). Full-text articles passing screening were assessed for relevance by all authors. Because this is a structured narrative review rather than a systematic one, selection bias cannot be fully ruled out. We attempted to mitigate this by conducting comprehensive multi-database searches. All neurobiological propositions in Section 3 are explicitly framed as hypotheses that require prospective empirical validation. All figures in this review are original works prepared by the authors from the data described in the text and in Supplementary Methods S1. No figure has been reproduced or adapted from a previously published article, website, or other external source, so no reproduction permission is required. The data underlying each figure are attributed in the corresponding legend and itemized in Supplementary Methods S1, Table S3.

Claude (Opus 4.8, Anthropic) was used for limited language editing, initial literature and reference searches, and initial diagram generation through author-written Python (Matplotlib) and HTML/CSS; all data and scientific interpretations were provided and verified by the authors, and each figure was subsequently reviewed and modified individually by the authors.

## 3. The Human–Animal Bond: Neurobiological Foundations and What Happens When It Breaks

Most reviews treat the neuroscience of bonding and the neuroscience of grief as separate topics. We regard this separation as a conceptual shortcoming. The very systems that sustain the bond are the ones whose disruption drives the grief. In the subsections that follow, we therefore pair each neurobiological substrate with the hypothesized consequences of its loss.

**Figure 2:**
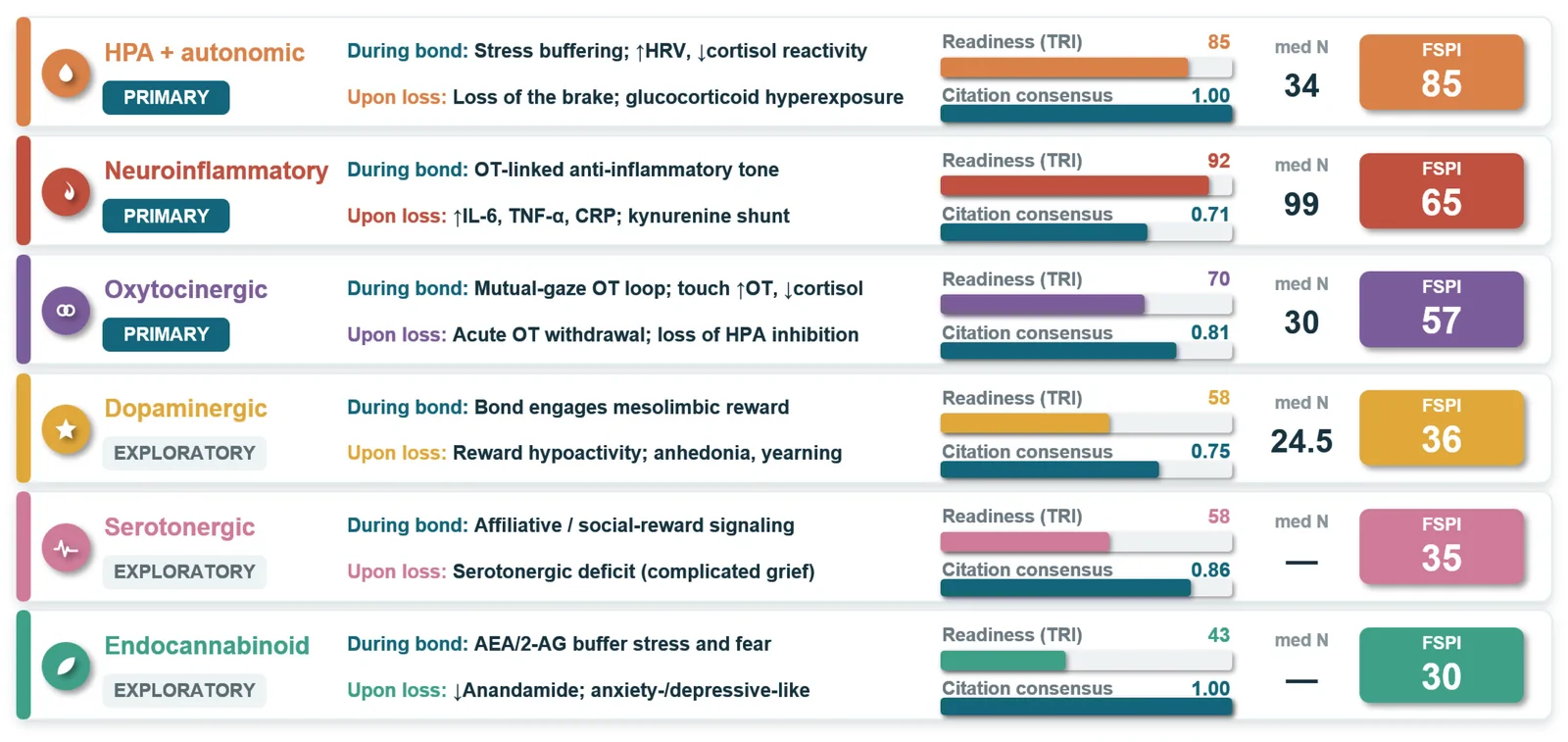
Mechanism map of the six candidate neurobiological systems whose disruption is hypothesized to drive pet loss grief, ranked by the First-Study Priority Index (FSPI). Systems are organized into a primary tier (oxytocinergic system, HPA axis, neuroinflammatory response), ready for first-generation biomarker testing, and an exploratory tier (dopaminergic, serotonergic, and endocannabinoid pathways), with the tier marked on each row. Each row summarizes the system’s role during the bond and its predicted change upon loss, and reports the analogical readiness index (TRI, 0–100), the citation-consensus ratio (0–1, from Scite Smart Citations), the median empirical sample size of the primary studies (N, shown as a dash where review-only), and the composite FSPI (=TRI × consensus × sample-adequacy). The color assigned to each system is the same as in Figure 1. Definitions, weights, and sensitivity analyses for the TRI and the FSPI are given in Supplementary Methods S1. Data sources: all index values are derived from the studies listed in Appendix A, Table A1, with the derivation of each value itemized in Supplementary Methods S1, Table S3.

One point governs the interpretation of everything that follows in this section. With the single exception of explicitly labeled predictions, all evidence cited below comes from human social bonding, human bereavement after human loss, or animal models. None of it comes from bereaved pet owners. The human and animal findings serve as scaffolding for hypotheses, not as confirmation of them. We organize the six systems into two tiers. Three carry comparatively strong analogical support and are ready for first-generation biomarker testing, namely the oxytocinergic system, the HPA axis with its autonomic partners, and the neuroinflammatory response. Three are more exploratory and rest on thinner evidence, namely dopaminergic reward circuitry and the serotonergic and endocannabinoid pathways. (Figure 2) Because no direct pet-loss biomarker data yet exist, every index value reported below is derived from analogical human and animal evidence, and the indices themselves are proposed heuristics rather than validated psychometric instruments.

### 3.1. The Oxytocinergic System (Primary)

Of all the neurobiological substrates of the human–animal bond, oxytocin has attracted the most sustained research attention. This nine-amino-acid neuropeptide is synthesized in the paraventricular and supraoptic nuclei, released from the posterior pituitary, and sits at the intersection of maternal behavior, pair bonding, social recognition, trust, and stress regulation [20]. Its deep evolutionary conservation across mammals indicates that social attachment is better understood as a biological necessity than as a sentimental luxury [21]. The broader psychosocial and psychophysiological effects of human–animal interaction, with oxytocin as a probable mediator, have been reviewed in detail [22].

The signature finding in this literature is bidirectional oxytocinergic activation. Early work showed that a dog’s gaze at its owner raises the owner’s urinary oxytocin [23]. Nagasawa and colleagues [24] went further, demonstrating in 2015 that mutual gaze between dogs and owners elevates urinary oxytocin in both species, an interspecific positive feedback loop without clear precedent. Oxytocin concentrations correlated with gaze duration and self-reported attachment, consistent with a dose–response relationship. Later studies confirmed that exogenous oxytocin enhances canine bonding behavior [25] and that tactile contact with a companion dog raises salivary oxytocin while lowering cortisol, blood pressure, and heart rate [26]. Oxytocin also modulates stress reactivity through direct inhibitory projections to the HPA axis, attenuating corticotropin-releasing hormone secretion from the paraventricular nucleus and suppressing adrenocorticotropic hormone release [27], and it facilitates fear extinction through actions on the amygdala [28].

A methodological caveat is warranted before these findings are extended to the pet loss context. Studies of human-dog oxytocin relationships have used heterogeneous biological matrices, namely urine, saliva, and plasma, and the correlations between matrices are weak [29]. The claim that companion-animal interaction sustains tonic oxytocin signaling therefore rests on convergent rather than directly comparable evidence. Future prospective studies should standardize the measurement modality and, where feasible, favor central over peripheral indices.

OXTR gene polymorphisms, particularly rs53576, have been proposed as moderators of social bonding and affiliative behavior [30]. A word of caution is in order before these polymorphisms are carried into the pet loss context. The candidate gene literature for rs53576 suffers from inconsistent replication, small effect sizes, and what appears to be substantial publication bias [31]. Treating these associations as straightforwardly applicable to pet loss grief without genome-wide approaches would be premature. The OXTR locus remains a biologically plausible exploratory target, but candidate-gene-era findings alone cannot support strong claims.

Hypothesized consequences of bond disruption, and the studies that would test them. During an active relationship, daily touch, eye contact, and olfactory exposure likely sustain elevated baseline oxytocin tone, which, in turn, inhibits HPA activity and supports autonomic homeostasis [26,32]. When the animal dies, the sudden and permanent removal of these oxytocin-stimulating interactions may precipitate an acute drop in oxytocinergic signaling, a collapse analogous to what happens when a rodent pair bond is experimentally disrupted. In prairie voles, breaking established bonds reduces oxytocin receptor binding in limbic structures and produces anxiety-like behavior and HPA hyperactivity [33]. Bereaved individuals show dysregulated diurnal cortisol patterns, flattened slopes, and altered awakening responses that persist for weeks to months [34]. From this reasoning, we derive a specific and testable prediction, namely that bereaved pet owners should show reduced oxytocin concentrations in acute bereavement and that these reductions should track grief severity and pre-loss attachment strength. This prediction has not yet been tested in bereaved pet owners.

### 3.2. The HPA Axis and Autonomic Nervous System (Primary)

Interaction with companion animals consistently down-regulates the stress axis. Pet owners show lower cortisol responses to psychosocial stressors than non-owners [35] and a systematic review encompassing 129 human–dog interaction studies confirmed cortisol reduction as one of the most robust findings in the field [36]. In certain experimental paradigms, the animal buffered stress more effectively than a spouse [37], a result that invites reflection on whether the non-evaluative, unconditional character of the human–animal relationship may offer stress-regulatory benefits that even close human relationships cannot fully replicate.

Heart rate variability, a reliable index of parasympathetic tone, increases during and after human-dog interaction [36,38]. Polyvagal theory [39] offers a complementary view, in which a trusted attachment figure activates the ventral vagal complex and promotes calm engagement. At the population level, pet ownership is associated with lower cardiovascular morbidity and mortality [40], likely reflecting sustained autonomic adaptation.

Hypothesized consequences of bond disruption, and the studies that would test them. With the stress buffer removed, the HPA axis loses its principal brake, so glucocorticoid hyperexposure is the predicted consequence. Here too the supporting evidence comes from human loss rather than from pet loss. Chronic grief after human bereavement is associated with sustained HPA activation and a dysregulated cortisol diurnal rhythm [41]. Elevated glucocorticoids suppress lymphocyte proliferation and natural killer cell cytotoxicity, impair hippocampal neurogenesis through reduced brain-derived neurotrophic factor, fragment sleep architecture, and promote systemic inflammation through NF-κB pathway interactions [42]. McEwen’s concept of allostatic overload [43] is useful here. Owners who have relied chronically on the bond’s stress-buffering effects may accumulate allostatic burden quickly when that resource disappears overnight. Whether any of this holds for pet loss specifically is unknown, and serial cortisol sampling in bereaved owners is the obvious first test.

### 3.3. Dopaminergic Reward Circuitry (Exploratory)

Social bonds engage the mesolimbic dopamine system, the circuitry responsible for reward prediction, motivational salience, and incentive learning. Bond dissolution lowers dopamine signaling in reward-related regions [44]. In a small human neuroimaging study, women with complicated grief showed greater nucleus accumbens activation in response to cues of the deceased than women with noncomplicated grief; activation was positively associated with yearning [45]. A separate study identified a distributed neural network involved in grief, including regions associated with affect processing, memory, and autonomic regulation [46]. These findings concern human bereavement and do not directly measure dopamine signaling after pet loss. Oxytocin and dopamine interact in the nucleus accumbens during bond formation [47], creating self-reinforcing loops that may heighten grief vulnerability when the bonded partner dies. Whether analogous dopaminergic changes occur in pet loss grief is unknown, and this gap is addressable.

Hypothesized consequences of bond disruption, and the studies that would test them. By analogy with human neuroimaging, pet loss may involve disruption of dopaminergic reward circuitry. Grief regulation engages prefrontal and limbic circuits [48], and the deceased animal may continue to act as a conditioned reward stimulus that drives the yearning and searching of acute grief. When these reward-seeking behaviors persistently go unreinforced, the plausible outcome is reward-system hypoactivity, expressed as anhedonia and social withdrawal [49]. Direct neuroimaging is needed before any of this can be asserted.

### 3.4. Serotonergic and Endocannabinoid Systems (Exploratory)

Serotonin contributes to affiliative behavior and social reward processing [50]. Endogenous opioids modulate social bonding as well, and on this point the animal evidence is direct. Panksepp and colleagues [51] showed that endorphins mediate the rewarding aspects of social contact and that disrupting opioidergic signaling produces separation distress in animal models. Central serotonergic activity falls in bereaved individuals with complicated grief and major depression [52].

The endocannabinoid system modulates social bonding, fear extinction, and stress resilience [53]. Anandamide and 2-arachidonoylglycerol act on CB1 and CB2 receptors to attenuate HPA responses, reduce amygdala-mediated fear, and support hedonic tone. In animal models, social separation lowers anandamide in the prefrontal cortex and amygdala and produces anxiety-like and depressive-like behaviors that pharmacological enhancement of the system can reverse [54].

Hypothesized consequences of bond disruption, and the studies that would test them. Neither serotonergic nor endocannabinoid changes have been investigated in pet loss grief, and the indirect evidence is thinner than for the primary systems. Serotonergic deficits are well established in complicated human grief and concurrent depression [52], and it would be surprising if pet loss grief of comparable severity did not involve similar disruption. The endocannabinoid data are thinner still, resting almost entirely on rodent separation paradigms. If social separation lowers prefrontal and amygdalar anandamide in rodents [54], prolonged absence of a bonded companion could plausibly produce analogous effects in humans, but this is conjecture. We place both systems in the exploratory tier and recommend that serotonergic and endocannabinoid assays be included as secondary measures in the biomarker panels of Section 8, valuable for hypothesis generation rather than confirmation.

### 3.5. Neuroinflammatory Cascades (Primary)

Social loss activates microglia and upregulates pro-inflammatory cytokines, including interleukin-1β, interleukin-6, and tumor necrosis factor-α, in limbic and prefrontal regions in animal models [55]. In humans, bereavement raises circulating inflammatory markers that predict grief severity and subsequent depression [56]. Some of this response may reflect withdrawal of the anti-inflammatory signaling normally provided by oxytocin [57].

**Figure 3:**
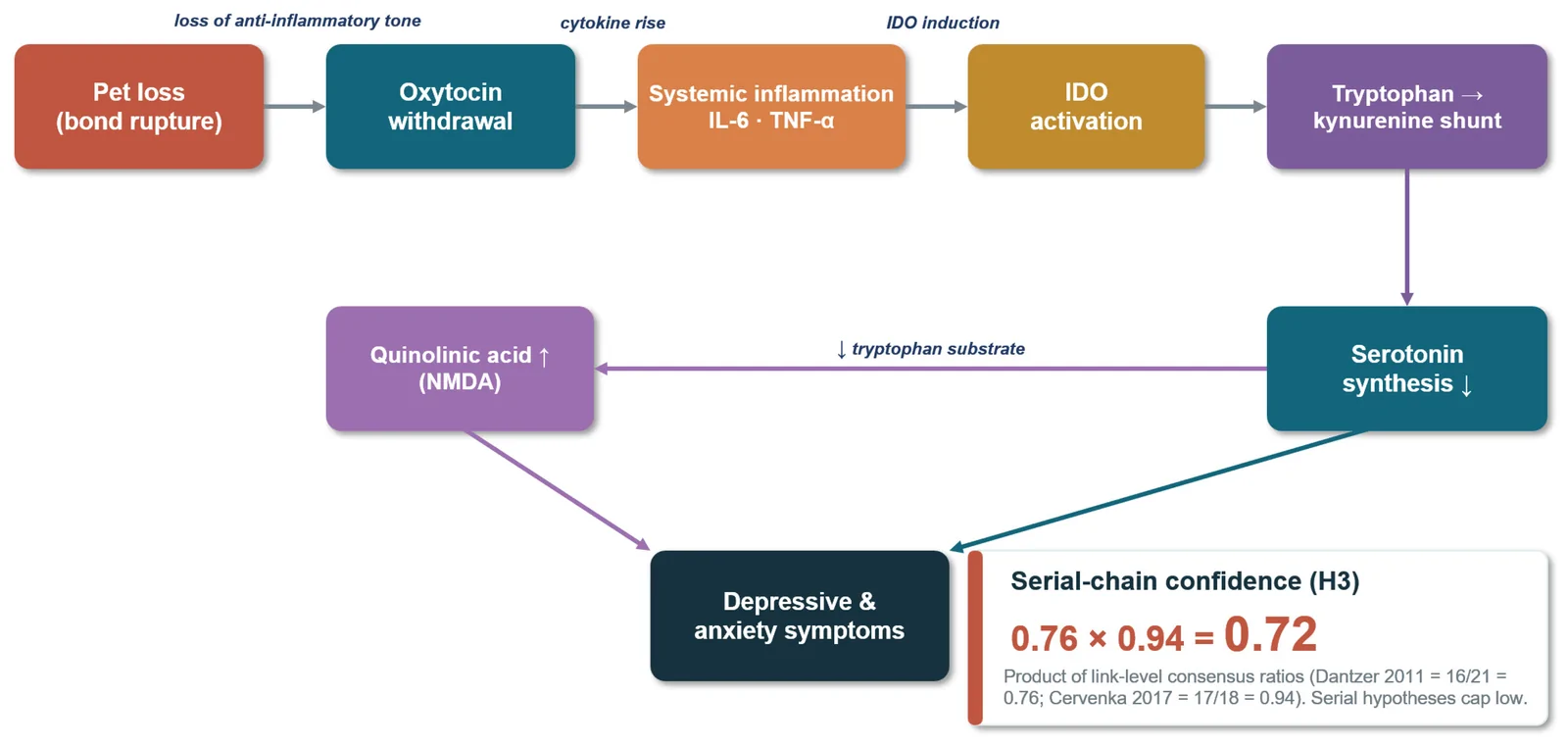
Putative kynurenine-pathway bridge linking peripheral inflammation to serotonergic deficit. Pro-inflammatory signaling induces indoleamine 2,3-dioxygenase (IDO), which shunts tryptophan away from serotonin synthesis and toward kynurenine and its neuroactive metabolite quinolinic acid. Link-level citation-consensus ratios (inflammation → kynurenine, 0.76, Dantzer et al. [59]; kynurenine → serotonin, 0.94, Cervenka et al. [60]) yield a serial-chain confidence of 0.72 (0.76 × 0.94). Data sources: the two link-level ratios are those of Dantzer et al. [59] and Cervenka et al. [60] as cited above, with derivations itemized in Supplementary Methods S1, Table S3.

Hypothesized consequences of bond disruption, and the studies that would test them. Neuroinflammation may drive depressive symptoms indirectly through tryptophan catabolism along the kynurenine pathway [58]. The proposed sequence begins when bereavement triggers a peripheral and central inflammatory response. Pro-inflammatory cytokines activate indoleamine 2,3-dioxygenase, which shunts tryptophan away from serotonin synthesis and toward neurotoxic kynurenine metabolites (Figure 3). The resulting serotonergic deficit and accumulation of quinolinic acid could sustain depressive and anxiety symptoms beyond what the psychological loss alone would predict. None of these pathways has been examined in bereaved pet owners. Given the robust association between human bereavement and inflammatory markers [56], and the plausible withdrawal of oxytocin-mediated anti-inflammatory protection upon pet death [57], inflammatory profiling of bereaved owners is a tractable and high-priority target. On the readiness dimension, the neuroinflammatory system ranks in the primary tier, but the data-driven robustness analyses in Section 3.7 identify it as the most fragile of the primary mechanisms. Its high readiness should therefore be read together with that qualification. A first direct test would pair serial inflammatory markers with grief severity in the same bereaved owners over the first months after loss, and the panel we would prioritize for that purpose is set out in Section 8.

### 3.6. Summary of Evidence and Critical Research Gaps

Table 1 maps each proposed mechanism against its evidence base and the studies needed to test it. The pattern is consistent across all six systems. Indirect or analogical evidence is available, but direct pet-loss-specific evidence is absent.

Specificity of the proposed markers and the problem of psychiatric comorbidity. None of the six markers considered above is specific to bereavement, still less to the loss of a companion animal. Altered diurnal cortisol rhythms, reduced oxytocinergic tone, blunted striatal reward signaling, and raised interleukin-6 and C-reactive protein are reported in major depressive disorder, in anxiety and post-traumatic stress disorders, and in chronic psychosocial stress of many kinds, and they accompany human bereavement in general rather than pet bereavement in particular. Two consequences follow for interpreting future studies. The first is inferential, in that a biomarker profile obtained after the death of a companion animal cannot by itself show that the death produced it, which is why the prospective pre-loss to post-loss design described in Section 8, in which each participant serves as their own baseline, is preferred to cross-sectional comparison. The second concerns concurrent psychiatric morbidity and is the more difficult of the two. A substantial proportion of bereaved owners will carry a pre-existing or emergent depressive or anxiety disorder, and the biological signature of such a disorder overlaps almost entirely with the signature predicted here for grief itself. To our knowledge, no study has characterized these systems in individuals experiencing pet loss grief together with a diagnosed mental disorder, and this is an informative gap rather than a peripheral one, because it is precisely in the comorbid group that clinical need is greatest. We therefore recommend that future studies screen for depressive and anxiety disorders at every time point, treat symptom severity as a covariate and as a candidate moderator rather than as an exclusion criterion, and, where sample size permits, stratify the analysis into bereaved owners without a concurrent diagnosis, bereaved owners with one, and non-bereaved individuals carrying the same diagnosis. Only a design of this kind can separate what is attributable to the loss from what is attributable to the disorder that accompanies it.

### 3.7. A Data-Driven Prioritization of Mechanisms

The six systems reviewed above differ not only in their proposed relevance to pet loss but also in the maturity, reliability, and structure of the evidence supporting them. To move beyond a descriptive account and compare the evidence supporting potential first studies, we integrated the quantitative evidence assembled for this review (79 extracted evidence records, corresponding to 75 unique publications, with extractable design, sample size, and citation data). Four of the 79 records were subsequently identified as duplicate entries for the same publication, which is why Appendix A lists 75 studies, and the index values reported below were computed on the full 79-record set. We report both counts so that the difference is not mistaken for an inconsistency. We then used the extracted data to construct a small set of transparent prioritization metrics. The full evidence base is reproduced in Appendix A, Table A1. These metrics are proposed heuristics rather than established psychometrics, and we report them together with their limitations. Full definitions, scoring rules, weighting, and sensitivity analyses for these metrics are provided in Supplementary Methods S1. We aim to identify, based on current evidence, which mechanism is the most defensible first target for a direct biomarker study of pet loss grief.

We first defined a First-Study Priority Index (FSPI) that combines three independent dimensions of evidential strength. The first is analogical readiness, reflecting how well a mechanism is supported by human bereavement and human–animal bond research that can be extended to pet loss. The second is citation consensus, computed for each mechanism as the ratio of supporting to supporting-plus-contrasting Smart Citations aggregated across its key papers. The third is sample adequacy, indexed by the median empirical sample size of the primary studies underlying the mechanism. Because these three dimensions are drawn from methodologically distinct sources, namely literature synthesis, a citation-classification database, and per-study sample extraction, their combination is informative rather than redundant. The composite is a product of these three inputs, and the readiness index itself weights its evidence dimensions according to the review’s priorities, namely human-bereavement support, human–animal-bond support, and quantified effect size. Because these choices are analytic rather than empirically derived, the resulting order should be read as a directional guide to which mechanism to test first, not as a precise or validated ranking, and its robustness to alternative weightings and to expansion of the evidence base is examined in Supplementary Methods S1.

**Figure 4:**
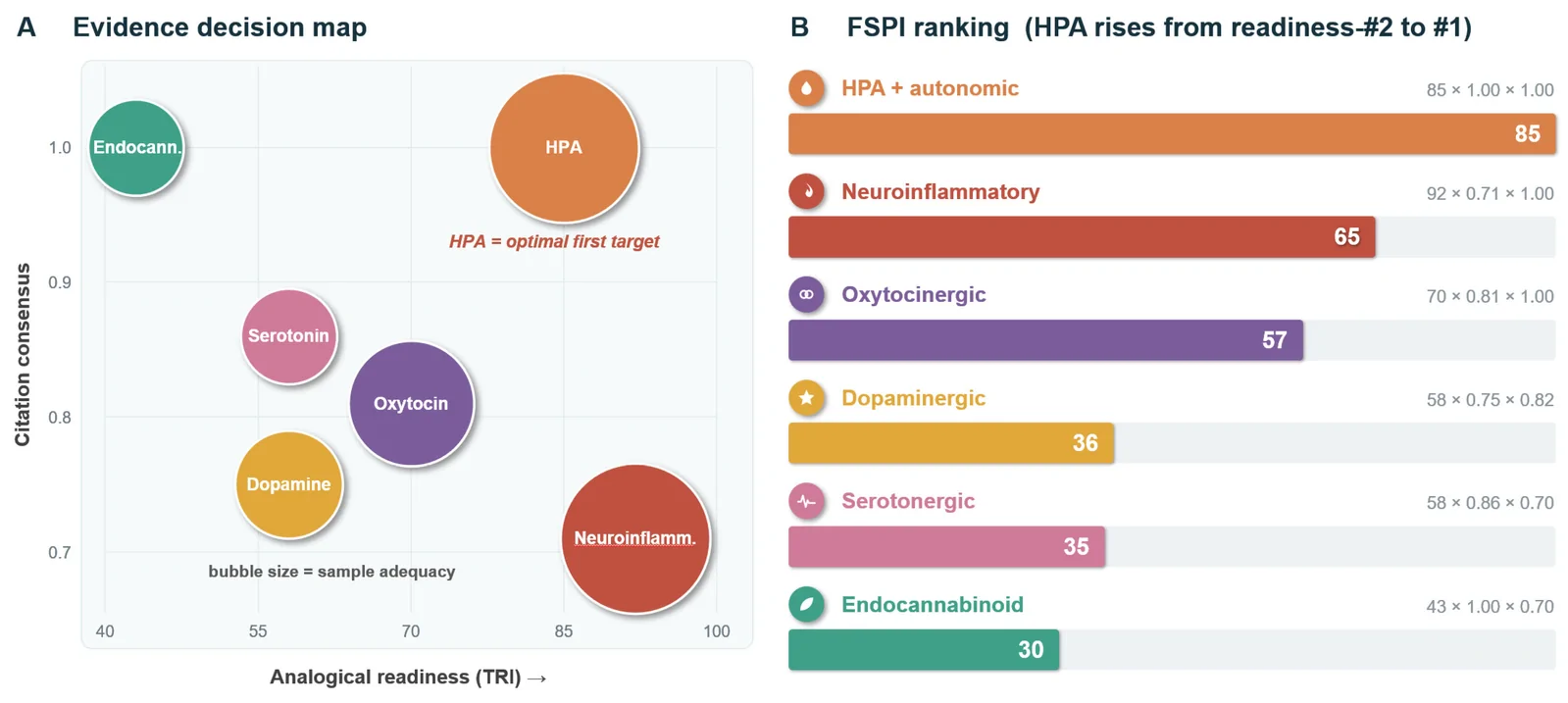
Data-driven prioritization of the six mechanisms by the First-Study Priority Index (FSPI). (**A**) Bubble plot showing analogical readiness (TRI), citation consensus, and sample adequacy for the six mechanisms. Bubble position encodes analogical readiness (*x*-axis, TRI) and citation consensus (*y*-axis). Bubble area encodes sample adequacy. (**B**) Bar chart showing the composite FSPI for the six mechanisms. Bars show the composite FSPI (HPA axis 85, neuroinflammatory 65, oxytocinergic 57, dopaminergic 36, serotonergic 35, endocannabinoid 30), each computed as TRI × consensus × sample-adequacy. The color assigned to each system is the same as in Figure 1 and Figure 2. Derivations are fully specified and reproducible in Supplementary Methods S1. Data sources: all index values are derived from the studies listed in Appendix A, Table A1, with the derivation of each value itemized in Supplementary Methods S1, Table S3.

The resulting ranking placed the hypothalamic–pituitary–adrenal (HPA) axis first, followed by the neuroinflammatory, oxytocinergic, dopaminergic, serotonergic, and endocannabinoid systems. This ordering is notable because it differs from what analogical readiness alone would suggest. On readiness alone, the neuroinflammatory system ranks highest, but when reliability and statistical power are incorporated, the HPA axis overtakes it. As a worked illustration, drawn from Table S1 of the Supplementary Materials, the HPA axis scores 85 × 1.00 × 1.00 = 85.0, whereas the neuroinflammatory system scores 92 × 0.71 × 1.00 = 65.1, so the stronger raw readiness of the neuroinflammatory system is outweighed once its lower citation consensus enters the product. The reordering proved stable across four sequential expansions of the evidence base (from 33 to 46 to 58 to 79 studies), and the HPA advantage widened rather than narrowed as evidence accumulated, because the neuroinflammatory literature acquired additional contrasting citations while the HPA literature did not. The HPA axis was ranked first in four of five alternative weighting schemes, indicating the result is not an artifact of a particular weighting choice (Figure 4).

Two further analyses, each methodologically independent of the FSPI and of the other, converged on the same conclusion. An evidence-triangulation analysis counted the number of distinct study designs supporting each mechanism (causal animal-loss model, human longitudinal, human experimental, cross-sectional, and synthesis review). The HPA axis was the only mechanism spanning four independent designs (Figure S1, Supplementary Materials), uniquely combining a causal animal-loss model (early maternal loss in wild chimpanzees) with human longitudinal and experimental evidence. By contrast, the neuroinflammatory and dopaminergic systems lacked a causal animal-loss anchor, resting solely on human observational and review evidence, which limits direct causal testing. A concentration analysis then quantified (Table S2 of the Supplementary Materials) how much of each mechanism’s citation weight rested on a single dominant paper. The neuroinflammatory system was the most concentrated, with roughly three-quarters of its citation weight attributable to one general review of relationships and inflammation that is not specific to bereavement. In contrast, the HPA and oxytocinergic systems drew on a more evenly distributed base of primary loss studies and therefore present no single point of failure.

**Figure 5:**
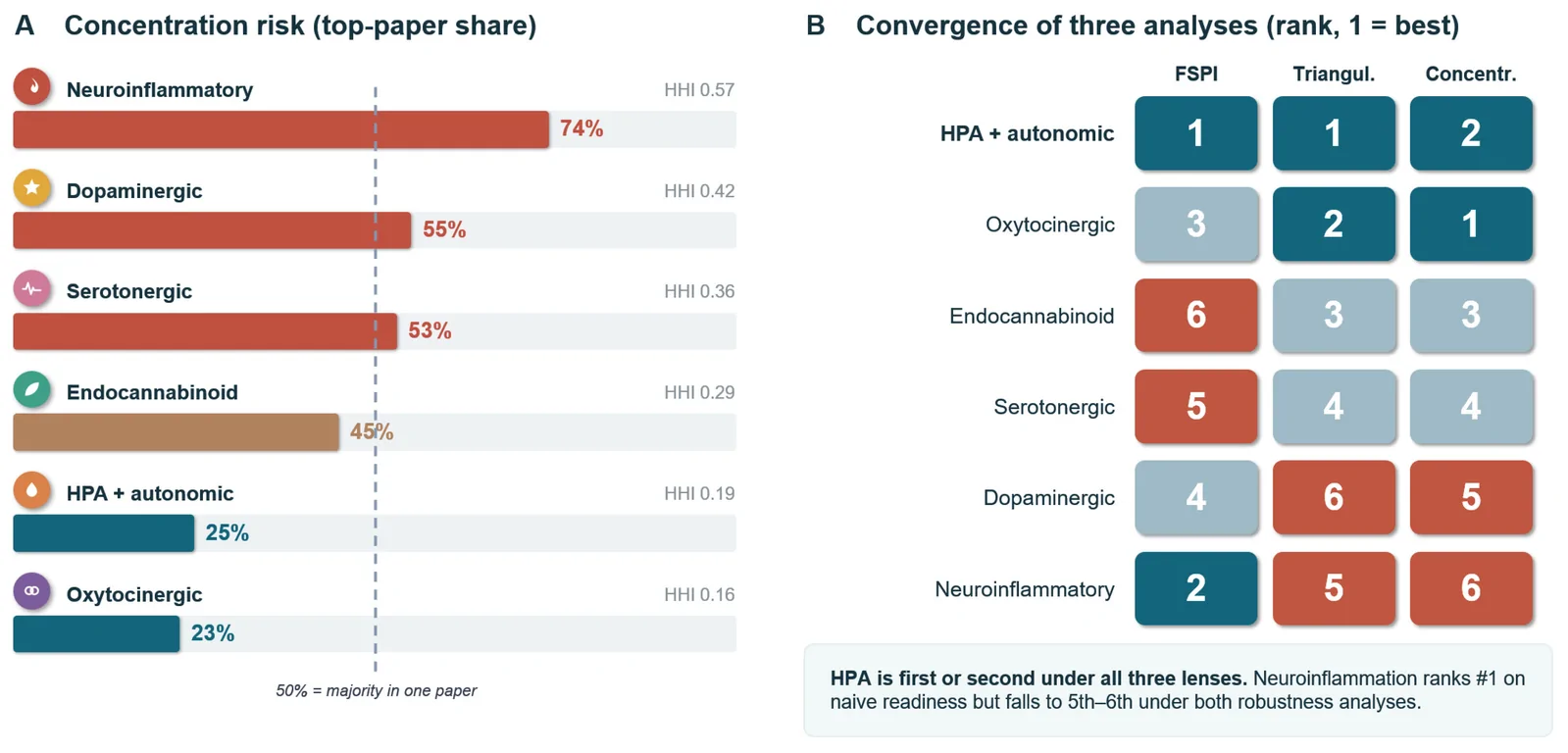
Robustness of the HPA-first ranking across independent lenses. (**A**) Citation-concentration analysis, using top-paper share and the Herfindahl index (HHI), identifies the neuroinflammatory system as the most single-source dependent (top-paper share ≈ 74%, HHI 0.57), whereas the HPA and oxytocinergic systems rest on a more evenly distributed base of primary loss studies. (**B**) Rank matrix showing the rank of each mechanism under the three methodologically independent analyses (FSPI, evidence triangulation, and citation concentration; 1 = best). See Supplementary Methods S1 for all derivations. Data sources: the concentration and triangulation analyses are computed from the citation records of the studies listed in Appendix A, Table A1. The color assigned to each system is the same as in Figure 1, Figure 2 and Figure 4.

The agreement of three independent analyses is the central result (Figure 5). The FSPI, the triangulation-breadth analysis, and the concentration analysis use different inputs yet all favor the HPA axis and all identify the neuroinflammatory system as comparatively fragile, since it is contested in its citation record, lacks a causal loss model, and is heavily dependent on a single non-specific review. The robustness of the HPA-first conclusion across these lenses gives us more confidence in it than any single metric would justify.

A complementary network analysis of how the six systems interconnect refines this recommendation. Mapping the mechanistic links reported across the extracted studies, the oxytocinergic and HPA systems emerged as the most integrative hubs (Figure S2, Supplementary Materials), while the neuroinflammatory system was the most peripheral, connected to the wider network only through the kynurenine pathway to serotonin. The single strongest link in the network was the coupling between oxytocin and cortisol. This suggests that an optimal first study should not measure the HPA axis in isolation but should assess the coupled HPA-oxytocin axis, since these two primary-tier systems are both individually robust and, mechanistically, most tightly linked.

Three boundary conditions must be stated plainly before these indices are put to use. First, a study-level analysis found that how contested a finding is could not be predicted from either its frequency of citation or its sample size, with both relationships statistically indistinguishable from zero. Citation consensus is therefore orthogonal to citation volume and statistical power, meaning popularity and sample size cannot substitute for direct evidence of agreement. Consensus is derived from automated Smart Citation classifications, which carry a nonzero misclassification rate, so we treat it as an approximate signal and rely on the agreement of independent analyses rather than on any single value. Second, and most importantly, every metric above is built on human bereavement and human–animal bond data, because direct biomarker studies of pet loss do not yet exist. These indices are intended to compare candidate mechanisms for an initial direct biomarker study and indicate where empirical testing might begin. They do not establish that any mechanism has been demonstrated in pet loss itself. Third, the dimension weights are the authors’ analytic choice rather than empirically derived. Although the ranking held under four of the five alternative weighting schemes tested and across successive expansions of the evidence base (Section S1.3 of the Supplementary Materials), the index should be interpreted as a directional priority rather than a precise ordinal scale. The prioritization is thus a hypothesis-generating tool, and the gap it addresses remains the central justification for the research priorities discussed in Section 8.

## 4. Pet Loss Grief: Clinical Manifestations, Epidemiology, and Psychosocial Context

### 4.1. Clinical Phenomenology

The clinical picture of pet loss grief spans affective, cognitive, somatic, and behavioral domains [1,61]. In many cases, it is indistinguishable from human bereavement, with sadness, depression, yearning, anxiety, guilt, anger, impaired concentration, intrusive memories, sleep disturbance, appetite changes, fatigue, and social withdrawal [62]. What sets it apart is not severity, which can be extreme, but social context. Bereaved pet owners often grieve without ritual, without institutional acknowledgment, and without private validation from those around them.

Among these features, guilt deserves particular attention. Park et al. [63] identified it as a prominent feature, most commonly arising from euthanasia decisions. The owner bears direct responsibility for the timing and manner of the animal’s death. This is a form of moral distress that has no close parallel in most human bereavements.

### 4.2. Epidemiology and Temporal Course

**Figure 6:**
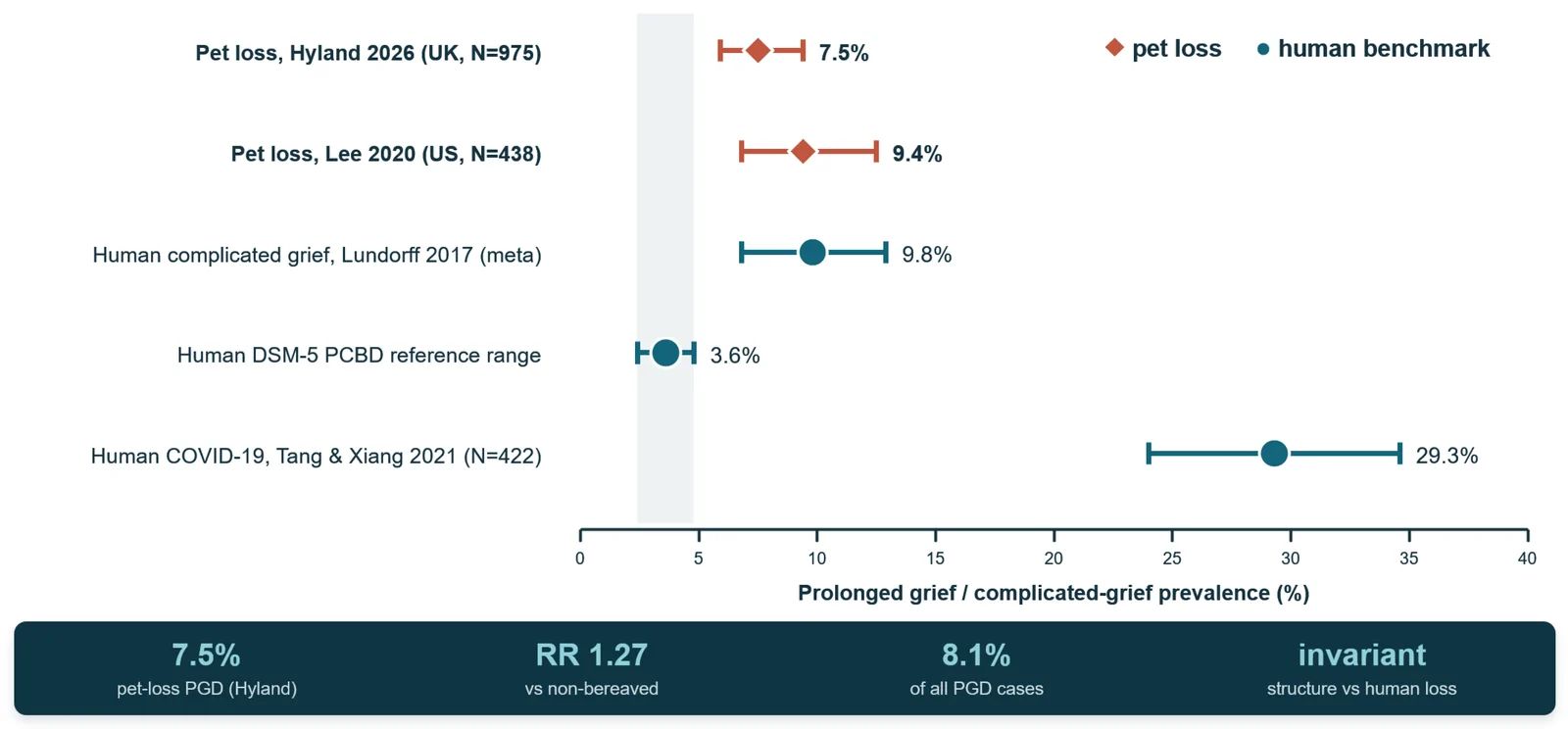
Prevalence of prolonged or complicated grief across bereavement contexts. Point estimates with reported intervals: pet loss 7.5% (95% CI 5.9–9.4; Hyland [10], N = 975) and 9.4% (Lee [2], N = 438); human complicated grief 9.8% (Lundorff [64], meta-analytic); a DSM-5 persistent complex bereavement reference midpoint of 3.6% (2.4–4.8% reference range); and COVID-19 bereavement 29.3% (Tang and Xiang [65], N = 422). Every point estimate is attributed to its source above, and the derivation of each value is itemized in Supplementary Methods S1, Table S3.

The duration of pet loss grief varies widely. Peer-reviewed evidence shows durations ranging from weeks to years, with attachment strength, coping resources, and the circumstances of death as major determinants of individual trajectories [17]. Hyland [10] reported that 7.5% of pet-bereaved individuals in a large UK sample met ICD-11 criteria for prolonged grief disorder, a prevalence comparable to many forms of human loss (Figure 6), and found that the symptom structure of prolonged grief did not differ between pet and human bereavements. Two caveats attach to this estimate. The first is that the sample was constructed through quota-based, non-probability methods rather than true random sampling, so it approximates rather than guarantees national representativeness. The second is that the estimate comes from a single Western study, so replication in other Western and East Asian samples is needed before any confident generalization. A further caution applies to the comparison shown in Figure 6, in which the estimates are not uniformly derived. The DSM-5 value is the midpoint of a reference range rather than a study estimate, and the human benchmark intervals are reported ranges rather than uniformly re-derived 95% confidence intervals. Lee [2] found that a meaningful subset of bereaved pet owners met DSM-5 criteria for persistent complex bereavement disorder, consistent with complicated-grief estimates in the general bereavement literature [64].

Risk factors include strong pre-loss attachment, inadequate social support, a ruminative coping style, single-person households, pre-existing mental health difficulties, and sudden or traumatic death [18,61,63,66]. Caring for a chronically ill pet before death has been separately linked to reduced quality of life and elevated stress [67]. Euthanasia is a risk factor in its own right, with higher decisional regret predicting worse outcomes [68].

### 4.3. Disenfranchised Grief and Its Cultural Dimensions

Doka’s [11] concept of disenfranchised grief captures why pet loss can be so psychologically corrosive. Several mechanisms drive it, in that the social environment trivializes human–animal relationships, mourning rituals are largely absent, bereavement leave is unavailable, and health professionals are reluctant to treat animal loss as clinically significant [69,70]. Emotional suppression, a common consequence of disenfranchisement, activates sympathetic pathways, elevates cortisol, impairs prefrontal regulation of amygdala reactivity, and raises the risk of somatic disease [71]. From a One Health perspective, this is precisely the kind of problem that goes unaddressed when veterinary and human health systems fail to communicate.

In Korean and broader East Asian settings, culturally shaped norms of emotional restraint may compound the universal disenfranchisement of pet loss [12,13]. Cross-cultural bereavement research shows that East Asian populations tend toward somatization, reduced verbal disclosure, and lower rates of mental-health help-seeking compared with Western populations [12,13]. Studies of Korean bereaved populations following human loss suggest that culturally shaped suppression may itself help maintain complicated grief reactions, although direct studies of Korean bereaved pet owners are absent. Recent work with Chinese university students showed that disenfranchised grief moderates the relationship between deliberate rumination and pet loss grief severity [72], which suggests that social non-recognition of pet loss interacts with maladaptive coping in at least this East Asian sample.

No one has yet directly compared pet loss grief between Korean and non-Asian populations. Without those data, we can only offer a hypothesis. The proposed mechanism is straightforward, in that the universal social trivialization of pet loss layers on top of East Asian norms of emotional restraint, and the predicted result is greater grief suppression, less help-seeking, and an elevated risk of complicated grief among bereaved Korean owners. We label this “double disenfranchisement”. To be clear, it is a working framework for testing, not a substantiated mechanism we are claiming to have established.

Two qualifications are warranted before this hypothesis is carried further. First, no published study has directly examined pet loss grief among Korean owners. The cross-cultural references invoked above involve Chinese, mixed East Asian, or general Asian samples rather than Korean populations specifically, so applying these findings to Korea is an inferential extension that, in turn, requires empirical validation. Second, to be properly falsifiable, the hypothesis must specify what would disconfirm it. We therefore state three deliberately specific falsification conditions, so that the hypothesis can be disconfirmed by data rather than merely appraised in qualitative terms. Korean owners do not differ from demographically matched Western owners in emotional suppression scores. Help-seeking rates do not differ after demographic adjustment. The prevalence of persistent complex bereavement disorder and prolonged grief disorder does not differ in the predicted direction. Effect sizes of at least 0.30 in the predicted direction would count as meaningful support, and effect sizes below 0.10 across all three predictions would count as disconfirmation. Whether the joint operation of universal trivialization and East Asian restraint is additive, multiplicative, or interactive should be modeled explicitly in cross-cultural designs. Within this framework, men may be especially vulnerable to self-disenfranchisement, the internalization of social norms that delegitimize one’s own grief [73].

### 4.4. Theoretical Models of Grief Applied to Pet Loss

Several established grief theories apply to pet loss without much modification. The Dual Process Model of Stroebe and Schut [74], which frames adaptive grieving as oscillation between loss-oriented and restoration-oriented coping, suits pet bereavement well, since getting stuck at either pole raises the risk of complicated grief. Neimeyer’s [75] meaning reconstruction framework highlights that companion animals serve as daily sources of routine, emotional anchoring, and relational meaning, so their death constitutes a genuine disruption of meaning. Worden’s [76] task-based model offers a more clinically actionable structure than the stage-based models that still circulate in popular culture, and the widely cited Kübler-Ross framework [77] in particular lacks empirical support. Continuing bonds also shape pet loss, as a systematic narrative synthesis found that maintaining psychological connection to a deceased animal can either ease or intensify grief depending on how the bond is held, with disenfranchisement tending to push it toward intensification [78].

## 5. Assessment Instruments

### 5.1. Existing Validated Instruments

Systematic assessment of pet loss grief requires validated, standardized instruments. Table 2 summarizes the principal measures currently available. One limitation applies across the board, in that none of these instruments has been formally validated in Korean-speaking populations. This is more than a methodological inconvenience, since it constitutes a substantial barrier to research in the region. A Chinese-language adaptation of the Pet Bereavement Questionnaire was validated in a Hong Kong Chinese sample in 2024 [79], but no Korean-language version exists.

### 5.2. Priorities for Culturally Adapted Tool Development in Korea

Korean-language instruments are necessary to examine the questions raised here empirically in Korean populations. Table S4 in the Supplementary Materials presents an illustrative sequence based on methodological dependencies.

## 6. Therapeutic Approaches

### 6.1. Evidence Classification Framework

A basic limitation of the therapeutic literature must be stated at the outset. No randomized controlled trial (RCT) has specifically targeted companion animal bereavement. Every psychotherapeutic recommendation, therefore, rests on evidence extrapolated from human bereavement research. Table 3 grades available approaches using a modified classification adapted from the Oxford Centre for Evidence-Based Medicine (CEBM) Levels of Evidence, with asterisked levels (e.g., Level II*) denoting extrapolated evidence derived from studies in analogous populations (e.g., human bereavement) rather than the target population (pet loss bereavement).

One entry in Table 3 warrants an added note. Intranasal oxytocin is rated Level V for its mechanistic plausibility from animal models and early human social-cognition studies. Yet, recent meta-analyses and methodological reviews have raised serious concerns about the reproducibility of intranasal oxytocin effects on human social and emotional outcomes [93,94]. The current evidence does not support clinical use. We include it only as a target for hypothesis-driven research, contingent on resolution of these methodological concerns.

### 6.2. The Role of Veterinarians

Veterinarians are often the first professionals a bereaved owner encounters after an animal’s death, and the quality of that encounter matters a great deal. Best-practice guidance stresses structured, empathic communication throughout end-of-life decision-making [95]. Empathic communication and collaborative decision-making reduce grief severity, lower decisional regret, and increase client satisfaction [68,96]. Communication failures predict worse grief outcomes, whether through rushed consultations, failure to acknowledge distress, or euphemistic language that obscures the reality of euthanasia [97].

This places veterinarians in an untenable position when they are not supported. Repeated exposure to animal death and owner grief drives compassion fatigue, and the profession faces elevated suicide risk [98]. Institutional support remains inadequate in most health systems, Korea included. From a One Health perspective, veterinary well-being is not a separate issue from owner bereavement, and the two are better regarded as different aspects of a single problem.

### 6.3. Interdisciplinary One Health Approaches

Full-scale integration across veterinary and mental health systems is logistically complex and unlikely to materialize as a single coordinated reform. Achievable near-term steps include standardized pet loss screening questions in primary care intake assessments; referral cards linking bereaved owners to grief support resources in veterinary clinic waiting rooms; shared continuing education that brings veterinary and mental health professionals into structured dialogue; and, where feasible, co-located or tele-linked counseling services accessible through veterinary clinics. A One Health framework does not require that every discipline solve every problem. It requires that each discipline recognize where its boundaries intersect with those of another and act accordingly.

## 7. Educational Implications for Healthcare Professionals

Pet loss grief is currently absent from veterinary, medical, and nursing curricula. This reflects an assumption, largely unexamined, that human–animal relationships are emotional rather than clinical concerns. We therefore propose a tiered interprofessional educational framework, addressed in turn to veterinarians, to physicians and psychiatrists, and to nurses.

Veterinarians need to recognize signs of grief, practice empathetic end-of-life communication, know how to use grief assessment tools, maintain current referral lists, and learn strategies to manage their own compassion fatigue. Veterinary schools should integrate these competencies into clinical rotations rather than relegating them to elective seminars.

Physicians and psychiatrists should be aware of DSM-5 diagnostic correlates, competent in screening for pet-loss-related depression and anxiety, and familiar with evidence-based therapeutic options. A patient who presents with depression after a pet’s death deserves the same diagnostic rigor as one who presents after any other significant loss.

Nurses are well-positioned to integrate companion animal ownership and pet loss history into routine assessments. An empathic, validating response from a nurse can be the first signal a bereaved patient receives that their grief is legitimate.

In Korea specifically, educational content must address the cultural norms around emotional restraint and the “double disenfranchisement” dynamic described in Section 4.3. Standardized patient simulations portraying bereaved Korean owners, with culturally authentic emotional restraint rather than open Western-style expressions of grief, would provide a valuable pedagogical tool for training Korean healthcare professionals. The goal is not to impose a Western grief model but to equip clinicians to recognize culturally encoded grief that manifests in indirect, somatized, or restrained forms.

## 8. Future Research Directions

The most pressing need is direct empirical testing of the neurobiological mechanisms proposed in Section 3. The priorities below are organized by methodological domain. The design features are illustrative and should be adapted to the study setting.

Biomarker studies. Prospective longitudinal studies could enroll participants before an anticipated loss and continue follow-up through bereavement. The coupled HPA-oxytocin axis is the primary candidate, assessed through concurrent diurnal cortisol and salivary oxytocin sampling. Inflammatory, endocannabinoid, and tryptophan/kynurenine measures could provide secondary outcomes. Studies should also assess attachment, grief severity, disenfranchisement, depressive and anxiety symptoms, and psychiatric comorbidity. Participants with concurrent diagnoses should be analyzed as clinically relevant subgroups rather than excluded.

Genetic moderators. Candidate gene approaches to OXTR rs53576 and 5-HTTLPR should be interpreted with extreme caution given the replication crisis in this literature [31]. Future genetic studies will require adequate statistical power and, where feasible, genome-wide approaches. Epigenetic analyses, including OXTR and NR3C1 methylation, offer a complementary and potentially more informative window into how bond disruption alters gene regulation.

Cross-cultural and qualitative research. Mixed-methods studies combining Korean-language qualitative interviews, validated Korean instruments, and cross-cultural comparisons could determine whether “double disenfranchisement” is specific to Korea or reflects a broader East Asian pattern.

Intervention trials. No intervention for pet loss grief has been evaluated in a randomized controlled trial. A pet-loss-adapted complicated grief treatment that addresses euthanasia-related guilt represents a logical initial test.

Pet loss grief research in East Asian populations remains limited. The Korean-language measures discussed in Section 5.2 would support biomarker, cross-cultural, and intervention studies in this population.

## 9. Conclusions

Pet loss grief deserves greater recognition from the biomedical and psychological research communities than it has received to date. The neurobiological framework we have proposed brings together oxytocinergic disruption, HPA dysregulation, dopaminergic reward-system hypoactivity, and neuroinflammation, but each piece is offered as a testable hypothesis rather than an established mechanism. The framework becomes actionable in its prioritization, since integrating analogical readiness, citation consensus, and sample adequacy identifies the HPA axis, assessed together with its coupled oxytocinergic partner, as the most defensible first target for a direct biomarker study. This ranking remained first under four of the five alternative weighting schemes tested, as the evidence base expanded from 33 to 79 studies. This ordering is a directional research priority, not a validated metric, and no mechanism has yet been measured directly in bereaved pet owners. We have been deliberate about several caveats throughout. Direct pet-loss-specific neurobiological evidence does not yet exist. The therapeutic recommendations rest on extrapolation from human bereavement research, and culturally adapted assessment instruments for East Asian populations still need to be built. One further limitation should be stated plainly, because no amount of hedging removes it. The case assembled here is analogical throughout, and analogical support, however internally consistent, cannot establish that the same biology operates when the deceased is a companion animal rather than a person. The proposed markers are moreover not specific to bereavement, let alone to pet bereavement, so any change observed in a bereaved owner will have to be distinguished from the effects of concurrent depressive and anxiety disorders. The framework should accordingly be judged by whether it generates studies capable of refuting it rather than by the strength of the analogies on which it currently rests.

With its growing companion animal population and distinctive cultural context, South Korea provides an important setting for research on culturally adapted assessment, neurobiology, and intervention.

Direct empirical studies are now needed to test the proposed framework. Prospective cohorts that begin before an anticipated pet loss could examine changes in oxytocin, diurnal cortisol, inflammatory markers, and endocannabinoids across bereavement. Pet-loss-specific intervention trials are also needed, with adapted complicated grief treatment addressing euthanasia-related guilt as one candidate. Testing the “double disenfranchisement” hypothesis will first require validated Korean-language assessment tools. These priorities define the main next steps without prescribing a fixed research program.

## Figures and Tables

**Table 1:** Evidence base, tier, and required empirical studies. None of the six neurobiological mechanisms has yet been examined directly in pet-bereaved populations. Every entry rests on analogical evidence and requires prospective validation, the gap this review addresses. The tier of each mechanism is also marked, so the strength of the underlying analogical case is visible at a glance.

Mechanism	Tier	Analogical Evidence	Required Studies
OT system disruption	Primary	Human–dog OT mutual gaze [24]; rodent pair bond disruption [33]	Prospective OT biomarker studies (saliva/urine); correlation with grief severity and pre-loss attachment
HPA dysregulation	Primary	Human bereavement cortisol elevation [34]; pet interaction cortisol reduction [36]	Serial cortisol (saliva, awakening response) in bereaved owners; wearable HRV monitoring
Dopaminergic disruption	Exploratory	Human complicated grief fMRI: greater cue-related nucleus accumbens activation [45]	fMRI or PET of pet-loss-specific yearning; longitudinal neuroimaging
Neuroinflammation	Primary	Human bereavement: elevated IL-6, TNF-α [56]; animal models [55]	Inflammatory biomarker panels (IL-6, CRP, TNF-α); correlation with grief severity
Serotonergic disruption	Exploratory	Human complicated grief: reduced 5-HT [52]	Tryptophan/kynurenine pathway assays; 5-HTTLPR genotyping as moderator
ECS dysregulation	Exploratory	Animal social separation: reduced AEA [54]	Plasma endocannabinoid assays (AEA, 2-AG); CB1/CB2 receptor imaging

**Table 2:** Principal instruments for pet loss grief assessment with East Asian validation status.

Instrument	Subscales/Items	Format	Clinical Application	East Asia Validation
Pet Bereavement Questionnaire (PBQ) [80]	Anger, Grief, Guilt (16 items)	4-pt Likert	Identify high-risk individuals; evaluate interventions	Not validated
Companion Animal Bonding Scale (CABS) [81]	Attachment strength (8 items)	5-pt Likert	Pre-loss risk stratification	Not validated
Pet Attitude Scale (PAS) [82]	Humanization, emotional closeness (13 items)	7-pt Likert	Characterize attachment style	Not validated
Inventory of Complicated Grief (ICG) [83]	Yearning, bitterness, shock (19 items)	5-pt Likert	Complicated grief screening	Korean version validated (ICG-K; see Note)
PHQ-9/GAD-7 [84,85]	Depression/Anxiety severity	4-pt Likert	Comorbid psychopathology screening	PHQ-9 validated in Korean
RBFM [86]	Decisional regret (5 items)	5-pt Likert	End-of-life care regret; euthanasia contexts	Not validated
CARE Measure [87]	Veterinarian empathy (10 items)	5-pt Likert	Veterinarian–client communication quality	Not validated

Note. The Korean version of the Inventory of Complicated Grief has been validated among Korean adolescents [88], and it is the only grief-specific tool listed here with formal Korean validation. Adult validation in pet bereavement contexts has not been established.

**Table 3:** Therapeutic approaches for pet loss grief graded by evidence level.

Intervention	Evidence Level	Supporting Evidence	Pet Loss RCT?	Recommended Use
Complicated Grief Treatment (CGT)	Level II*	RCT in human complicated grief [89]	No, extrapolated	First-line for complicated grief; pet-specific trial urgently needed
Cognitive-Behavioral Therapy (CBT)	Level II*	RCTs in human bereavement-related depression [90]	No, extrapolated	First-line for comorbid depression/anxiety; adapt for euthanasia guilt
Meaning-centered therapy	Level III*	Theoretical framework [75]; case series	No, extrapolated	Adjunctive: useful for existential dimensions of loss
Peer support/online groups	Level III*	Observational studies in pet loss [17,18]	No, observational only	Accessible; counteracts disenfranchisement; not standalone
SSRIs/SNRIs	Level II*	RCTs as augmentation in human complicated grief [89]	No, extrapolated	Consider for comorbid depression; not first-line for uncomplicated grief
Logotherapy	Level IV	Theoretical basis [91]; no controlled trials	No	Experimental; may benefit individuals with existential orientation
Intranasal oxytocin	Level V (speculative)	Animal models; human social bonding studies [92]	No, highly speculative	Research only; no clinical use at present

Evidence grading note: Level II* = evidence extrapolated from RCTs in analogous human bereavement populations; Level III* = observational evidence or expert consensus extrapolated to pet loss; Level IV = theoretical basis only; Level V = speculative/experimental. No Level I evidence (pet-loss-specific RCTs) currently exists.

## Data Availability

The data supporting the findings of this review are contained within the article, including the complete quantitative evidence base presented in Appendix A (Table A1). The full evidence database, comprising the extracted studies and the derived analytic sheets, is available from the corresponding author upon reasonable request.

## Supplementary Materials

The following supporting information can be downloaded at https://www.mdpi.com/article/10.3390/ani16193129/s1. Supplementary Methods S1, index definitions, scoring rules, weighting, and sensitivity and robustness analyses for the Translational Readiness Index and the First-Study Priority Index, together with the supporting tables (Table S1, base-scheme inputs and index ranking; Table S2, triangulation breadth and concentration risk; Table S3, figure-value provenance index) and a supplementary reference list; Figure S1, evidence triangulation architecture across the six mechanisms; Figure S2, mechanism co-occurrence network and centrality; Table S4, illustrative sequence for culturally adapted pet loss grief assessment in Korean and East Asian populations.

## Abbreviations

The following abbreviations are used in this manuscript:

2-AG	2-arachidonoylglycerol
5-HT	5-hydroxytryptamine (serotonin)
5-HTTLPR	serotonin-transporter-linked polymorphic region
AEA	anandamide (N-arachidonoylethanolamine)
CABS	Companion Animal Bonding Scale
CBT	cognitive behavioral therapy
CEBM	Centre for Evidence-Based Medicine
CGT	complicated grief treatment
CRP	C-reactive protein
DSM-5	Diagnostic and Statistical Manual of Mental Disorders, 5th Edition
ECS	endocannabinoid system
FSPI	First-Study Priority Index
GAD-7	Generalized Anxiety Disorder 7-item scale
HHI	Herfindahl-Hirschman Index
HPA	hypothalamic–pituitary–adrenal (axis)
HRV	heart rate variability
ICD-11	International Classification of Diseases, 11th Revision
ICG	Inventory of Complicated Grief
ICG-K	Inventory of Complicated Grief, Korean version
IDO	indoleamine 2,3-dioxygenase
IL-6	interleukin-6
OT	oxytocin
OXTR	oxytocin receptor gene
PAS	Pet Attitude Scale
PBQ	Pet Bereavement Questionnaire
PGD	prolonged grief disorder
PHQ-9	Patient Health Questionnaire-9
PRISMA	Preferred Reporting Items for Systematic Reviews and Meta-Analyses
RCT	randomized controlled trial
SANRA	Scale for the Assessment of Narrative Review Articles
TNF	tumor necrosis factor
TRI	Translational Readiness Index

## Appendix A

**Table A1:** Quantitative evidence base: the 75 unique studies extracted for the mechanism-prioritization analysis. N denotes sample size (“review” or “—” where not applicable); S/C/M denotes Scite Smart Citation tallies (Supporting/Contrasting/Mentioning), with “—” where not tallied.

#	Study	Journal	N	Design	Key Quantitative Finding	S/C/M	DOI
1	Hyland 2026 [10]	PLoS ONE	975	cross-sectional survey	Pet-loss PGD 7.5%; RR 1.27; pet loss = 8.1% of all PGD; full measurement invariance	—	10.1371/journal.pone.0339213
2	Lee 2020 [2]	Psychiatry Research	438	survey	9.4% met DSM-5 PCBD criteria after pet loss	—	10.1016/j.psychres.2020.112800
3	Yiu et al. 2024 [79]	Animals	246	psychometric validation	PBQ-C 3-factor (grief/anger/guilt); correlates with ICG & DASS-21 depression	0/0/0	10.3390/ani14192845
4	Park and Royal 2020 [99]	Veterinary Sciences	340	national survey	Euthanasia coping: 74.7% mourned privately, 58.2% social support, 32.1% new pet, 0.9% support group	0/0/8	10.3390/vetsci7030089
5	Testoni et al. 2017 [100]	Anthrozoos	1249	survey	Pet loss linked to death representation, attachment, depression, euthanasia attitudes	7/0/41	10.1080/08927936.2017.1270599
6	Field et al. 2009 [61]	Death Studies	—	survey	Attachment style predicts pet loss grief severity	4/1/102	10.1080/07481180802705783
7	Packman et al. 2011 [78]	J Loss & Trauma	—	survey	Continuing bonds associated with psychosocial adjustment in pet loss	6/0/76	10.1080/15325024.2011.572046
8	Ogi et al. 2020 [101]	Animals	8	experimental pilot	Salivary OT rose after positive interaction (*p* = 0.036); no HPA change	4/0/49	10.3390/ani10040708
9	Handlin et al. 2011 [26]	Anthrozoos	—	experimental	Owner–dog interaction alters OT, cortisol, insulin, HR (OT–cortisol inverse relationship contested)	12/6/260	10.2752/175303711X13045914865385
10	Nagasawa et al. 2017 [102]	Front Psychology	—	experimental	Intranasal OT increased dog-owner gaze; owner urinary OT rose; HRV changes	2/1/28	10.3389/fpsyg.2017.01624
11	Simpson et al. 2014 [103]	PNAS	28	RCT (macaque)	Inhaled OT decreased salivary cortisol (d = 0.99, *p* = 0.018)	5/2/60	10.1073/pnas.1402471111
12	Miller et al. 2022 [104]	Front Vet Science	—	review	Therapy-dog HAI raised salivary OT (*p* = 0.02; vs. baseline *p* < 0.01)	1/0/4	10.3389/fvets.2022.844252
13	Pedrinelli et al. 2025 [105]	Int J Mol Sci	12 studies	systematic review	Altered cortisol rhythm, elevated OT, raised cytokines across pathological grief (12 of 2140 screened)	—	10.3390/ijms262411835
14	Brown et al. 2022 [106]	Psychological Science	111	experimental	High grief: +45%/h IL-6 vs. +26%/h low grief (19% relative), independent of depression	—	10.1177/09567976211059502
15	Zuelke et al. 2021 [107]	JMIR Mental Health	1349	meta-analysis (9 RCTs)	Internet grief therapy: grief g = 0.54, depression g = 0.44, PTS g = 0.82	—	10.2196/29661
16	Lundorff et al. 2017 [64]	J Affective Disorders	meta	meta-analysis	Prolonged grief 9.8% of bereaved adults	—	10.1016/j.jad.2017.01.030
17	Ong et al. 2011 [34]	Health Psychology	44	prospective (bereaved vs. control)	Spousal loss: lower wake cortisol + flatter diurnal slope; positive emotion mediates 29% of slope effect	1/0/48	10.1037/a0022262
18	Hopf et al. 2020 [108]	J Neuroendocrinology	20 studies	systematic review (460 screened)	Elevated mean cortisol, flattened diurnal slopes, higher morning cortisol in bereaved	0/0/21	10.1111/jne.12887
19	Seiler et al. 2020 [109]	Front Psychiatry	review	conceptual review	Within 6 mo of spousal loss: HPA dysregulation + heightened inflammation + reduced NK activity	1/0/54	10.3389/fpsyt.2020.565239
20	Girard-Buttoz et al. 2021 [110]	eLife	78	wild chimpanzee (animal loss model)	Maternal loss raised HPA activation (diurnal slope) short-term in immatures; recovered in adults	1/0/33	10.7554/elife.64134
21	Kakarala et al. 2020 [111]	Psychiatry Res Neuroimaging	24 studies	systematic review (reward system)	PGD shows differential NAc/amygdala/OFC/ACC activity; 15 of 18 fMRI studies had N < 30	3/1/44	10.1016/j.pscychresns.2020.111135
22	O’Connor et al. 2008 [45]	NeuroImage	23	fMRI (PGD vs. normative)	Higher nucleus accumbens activation to deceased cue in PGD (N = 11) vs. normative grief (N = 12)	—	10.1016/j.neuroimage.2008.04.256
23	Bryant et al. 2021 [112]	Psychological Medicine	117	fMRI (PGD/PTSD/MDD/control)	PGD had distinct medial OFC + reward-region activation vs. PTSD and MDD	0/0/26	10.1017/s0033291719003507
24	McConnell et al. 2018 [113]	Heliyon	25	fMRI	Yearning predicted subgenual ACC activation across bereaved (reward/rumination)	0/0/12	10.1016/j.heliyon.2018.e00852
25	Bui et al. 2019 [114]	Eur J Psychotraumatol	—	case–control	Circulating oxytocin higher in PGD than non-PGD (peripheral; caution re central)	—	10.1080/20008198.2019.1646603
26	Dantzer et al. 2011 [59]	Psychoneuroendocrinology	review	mechanistic review	Inflammation shifts tryptophan from serotonin to kynurenine via IDO; neurotoxic 3-HK/quinolinic acid drive depression	16/5/519	10.1016/j.psyneuen.2010.09.012
27	Correia and Vale 2022 [115]	Int J Mol Sci	review	narrative review	~95% of free tryptophan enters kynurenine pathway; IDO/TDO overactivated in depression; 5-HT reduced	1/0/169	10.3390/ijms23158493
28	Cervenka et al. 2017 [60]	Science	review	mechanistic review	Peripheral inflammation raises brain kynurenine (depression); exercise clears it via skeletal muscle	17/1/1137	10.1126/science.aaf9794
29	Wei et al. 2016 [116]	Cannabis Cannabinoid Res	animal	experimental (mouse)	Oxytocin drives anandamide-mediated CB1 signaling in NAc to control social reward; FAAH block reverses social deficit	6/0/120	10.1089/can.2015.0008
30	Chen et al. 2011 [117]	PNAS	194	experimental (G × E)	OXTR rs53576 G-allele carriers show lower cortisol to stress after social support; AA genotype no buffering	19/0/213	10.1073/pnas.1113079108
31	Onaka and Takayanagi 2021 [118]	J Neuroendocrinology	review	review	Maternal separation alters OXTR binding (lateral septum down, VMH up); early stress reshapes oxytocin system	0/0/60	10.1111/jne.13049
32	Romero et al. 2014 [25]	PNAS	behavioral	experimental (dog)	Exogenous OT raised affiliation/social orientation to owners & dog partners; contested (4 contrasting)	5/4/197	10.1073/pnas.1322868111
33	Wirobski et al. 2021 [119]	Scientific Reports	dogs+wolves	experimental	Pet-dog OT correlated with owner physical contact; life experience not domestication drives OT release	1/0/21	10.1038/s41598-021-93922-1
34	Payne et al. 2015 [120]	Psych Res Behav Manag	review	review	Affiliative dog–human interaction produces mutual physiological change (OT up, cortisol down)	6/0/176	10.2147/prbm.s74972
35	Thielke and Udell 2017 [121]	Biological Reviews	review	review	Intranasal OT proposed for canine separation anxiety; role of OT in dog-human bond	0/0/23	10.1111/brv.12235
36	Oliva et al. 2016 [122]	Pet Behaviour Science	75	experimental (crossover)	Intranasal OT altered dog object-choice performance; owner attachment predicted saline not OT trials	0/0/13	10.21071/pbs.v0i1.3991
37	Hattori et al. 2024 [123]	Scientific Reports	30	experimental (cat)	Intranasal OT increased gaze to humans in male cats (sex-specific); interspecies OT effect	0/0/2	10.1038/s41598-024-59161-w
38	D’Aniello et al. 2022 [124]	Animal Cognition	behavioral	experimental (SST)	Sex differences in dog-owner attachment parallel adult human romantic bonds	1/0/5	10.1007/s10071-021-01545-w
39	O’Connor et al. 2012 [125]	Psychoneuroendocrinology	24	case–control	Complicated grief (n = 12) had flatter diurnal cortisol slope than non-complicated (n = 12)	1/0/38	10.1016/j.psyneuen.2011.08.009
40	Mason and Duffy 2019 [126]	J Am Psychiatr Nurses Assoc	review	integrative review	Complicated grief associated with altered cortisol response; HPA dysregulation	0/0/18	10.1177/1078390318807966
41	Guldin et al. 2011 [127]	BMC Palliative Care	276	cohort	BDI + single item at 8 wk screens CG risk (AUC 0.83); stratified 7%/23%/64% propensity	2/2/32	10.1186/1472-684x-10-9
42	Breen et al. 2020 [128]	Palliative Medicine	70	prospective longitudinal	Caregiver grief/QoL/health took 9–10 mo post-death to match non-caregiver comparison	4/0/42	10.1177/0269216319880766
43	Mah et al. 2022 [129]	Support Care Cancer	157	cohort	Death preparation predicted less complicated grief (beta = −0.37, *p* = 0.001) and depression (beta = −0.35, *p* = 0.005)	2/0/20	10.1007/s00520-021-06536-8
44	Peak et al. 2026 [130]	OMEGA	304	cross-sectional	Maladaptive personality + coping predicted complicated-grief symptoms; coping partially mediated	0/0/0	10.1177/00302228241275195
45	Fagundes et al. 2019 [131]	Psychoneuroendocrinology	99	case–control	Bereaved showed higher stimulated proinflammatory cytokines (IL-6, TNF-α, IFN-γ, IL-17A); depression predicts	2/0/60	10.1016/j.psyneuen.2018.10.006
46	Schultze-Florey et al. 2012 [132]	Brain Behav Immun	—	case–control (G × E)	Bereaved vs. married: inflammatory response varies by IL-6 genotype; genetic moderation of grief inflammation	3/1/51	10.1016/j.bbi.2012.06.009
47	LeRoy et al. 2020 [133]	Psychoneuroendocrinology	—	case–control	Attachment orientation moderates bereavement inflammation (IL-6, fibrinogen, CRP)	2/1/15	10.1016/j.psyneuen.2019.104401
48	Kiecolt-Glaser et al. 2010 [134]	Neurosci Biobehav Rev	review	review	Close relationships modulate inflammation and health; relationship disruption raises inflammatory risk	10/5/362	10.1016/j.neubiorev.2009.09.003
49	Mann 2013 [135]	Phil Trans R Soc B	review	review	Serotonergic dysfunction central to mood disorders and suicidal behavior; 5-HT deficit + receptor changes	6/1/136	10.1098/rstb.2012.0537
50	Mann and Currier 2010 [136]	Eur Psychiatry	review	review	Stress-diathesis: serotonergic and HPA alterations plus epigenetics underlie suicide neurobiology	2/0/101	10.1016/j.eurpsy.2010.01.009
51	Gibson 2018 [137]	Proc Nutr Soc	review	review	Tryptophan supplementation raises serotonin function; effects moderated by genetic variation	2/0/55	10.1017/s0029665117004451
52	Lamontagne et al. 2022 [138]	Neurobiol Stress	review	cross-species review	Stress-induced endophenotypes of suicide conserved across species (serotonergic/reward)	0/0/3	10.1016/j.ynstr.2022.100450
53	Fitzgerald et al. 2021 [139]	Neurobiol Stress	—	prospective	Circulating endocannabinoids (AEA/2-AG) prospectively predicted depression risk after trauma	2/0/18	10.1016/j.ynstr.2021.100304
54	Karhson et al. 2016 [140]	Translational Psychiatry	review	RDoC review	Endocannabinoid signaling regulates social functioning; anandamide-oxytocin interplay in social reward	1/0/47	10.1038/tp.2016.169
55	Bassir Nia et al. 2019 [141]	Chronic Stress	review	review	Endocannabinoid system altered in PTSD; AEA/2-AG dysregulation with trauma and stress	2/0/32	10.1177/2470547019864096
56	Maldonado et al. 2020 [142]	Dialogues Clin Neurosci	review	review	Endocannabinoid system modulates fear, anxiety, stress-coping; CB1 central to emotional homeostasis	1/0/49	10.31887/dcns.2020.22.3/rmaldonado
57	Sun et al. 2014 [143]	Behav Brain Res	animal	experimental (prairie vole)	Partner-loss in male prairie voles caused long-term emotional/neurochemical changes (OT/reward)	10/3/125	10.1016/j.bbr.2014.02.016
58	Love 2014 [144]	Pharmacol Biochem Behav	review	review	Oxytocin interacts with dopamine to drive motivation and social reward	11/0/249	10.1016/j.pbb.2013.06.011
59	Vitale and Smith 2022 [145]	Front Behav Neurosci	review	integrative review	Loneliness, isolation and loss share neurobiology of social-reward and stress systems	5/0/63	10.3389/fnbeh.2022.846315
60	Luyten and Fonagy 2018 [146]	Clin Psychol Rev	review	model review	Stress-reward-mentalizing model links depression to reward dysfunction and attachment	3/0/60	10.1016/j.cpr.2017.09.008
61	Pedersen 2004 [147]	Ann N Y Acad Sci	review	review	Oxytocin/vasopressin underpin social bonding and its disruption; roots of mother-infant attachment	3/0/73	10.1196/annals.1330.006
62	Kaplow et al. 2013 [148]	J Trauma Stress	—	cohort	Bereavement-related HPA axis dysregulation correlates with environmental and psychological factors	3/0/18	10.1002/jts.21788
63	Debbané et al. 2016 [149]	Front Hum Neurosci	review	review	Attachment neurobiology and mentalizing across the psychosis continuum (OT, mesolimbic)	4/0/117	10.3389/fnhum.2016.00406
64	Bonanno et al. 2004 [150]	Psychology and Aging	—	prospective longitudinal	5 bereavement trajectories (resilience most common); chronic grief vs. chronic depression distinct	32/5/356	10.1037/0882-7974.19.2.260
65	Vable et al. 2015 [151]	Am J Geriatr Psychiatry	8106	cohort (HRS)	Widowhood effect precedes loss; recent widows worse depression (beta = 0.71, 95%CI 0.57–0.85)	7/1/65	10.1016/j.jagp.2014.05.004
66	Ong et al. 2010 [152]	Psychology and Aging	—	prospective	Widowed showed decline in positive emotion vs. married; preloss resilience buffered	5/1/46	10.1037/a0018870
67	Stroebe and Schut 2001 [153]	APA (Handbook)	review	methodological review	Risk factors for poor bereavement outcome; dual-process model; early risk identification	6/2/99	10.1037/10436-015
68	Palitsky et al. 2023 [154]	Psychosomatic Medicine	59	experimental (grief recall)	Grief recall raised SBP +21.1 mmHg; PGD severity predicted SBP response (cardiovascular grief link)	3/1/35	10.1097/psy.0000000000001223
69	Jadhav and Weir 2018 [155]	J Gerontol B	—	cross-national cohort	Widowhood-depression link across US/Europe/Korea/China; gender-specific symptom patterns	4/1/58	10.1093/geronb/gbx021
70	Szabó et al. 2020 [156]	J Gerontol B	686	longitudinal (LCGA)	Emotional loneliness the most common post-bereavement change; 31% clinically relevant depression	6/0/41	10.1093/geronb/gbz039
71	Kamp et al. 2019 [157]	Death Studies	175	cross-sectional	Bereavement hallucinations linked to prolonged grief/PTSD/depression; avoidant coping predicts	8/1/39	10.1080/07481187.2018.1458759
72	Guiaux et al. 2007 [158]	Personal Relationships	635	prospective	Support rose after widowhood then declined ~2.5 yr later; network instability in late life	10/4/104	10.1111/j.1475-6811.2007.00165.x
73	Cohen-Mansfield et al. 2013 [159]	Psychol Trauma	1239	cohort (20-yr)	Parental bereavement predicted mortality at 20 yr; stronger for mothers	4/0/24	10.1037/a0029011
74	Shear and Mulhare 2008 [160]	Psychiatric Annals	review	review	Attachment-based model of acute/integrated/complicated grief; CG clinical syndrome + treatment	3/0/90	10.3928/00485713-20081001-10
75	Zhang et al. 2019 [161]	SSM Popul Health	—	cohort (CHARLS)	Continued widowhood 2+ yr predicts episodic memory decline in Chinese older adults	3/1/36	10.1016/j.ssmph.2018.100329
