# Supplementary material for: Neurobiology of Pet Loss Grief: A Review from a One Health Perspective
Source: Animals (Basel). 2026 Oct 6;16(19):3129. doi: 10.3390/ani16193129 (PMC13663545; doi:10.3390/ani16193129)
Supplement: Supplementary file 1 [file animals-16-03129-s001.zip › Supplementary_Materials_S1.pdf]

Supplementary Methods S1. Prioritization metrics (TRI, FSPI), robustness analyses, and figure-value provenance

This document specifies how the prioritization heuristics reported in Section 3.7 were computed and traces every quantitative value that appears in Figures 1–6 to its source and calculation, so that the ranking and the figures can be reproduced and their sensitivity to analytic choices assessed. The Translational Readiness Index (TRI) and First-Study Priority Index (FSPI) are proposed heuristics for a hypothesis-generating review, not validated psychometric instruments. Citation-consensus statistics were extracted from Scite Smart Citations; per-mechanism sample sizes and study designs were extracted from the evidence base compiled for this review. Author–year citations below are keyed to the reference list at the end of this document (Section S1.7).

Supplementary tables are numbered S1 to S4 in a single series. In the originally submitted supplement the first three were lettered S1a, S1b, and S1c, and they are now Tables S1, S2, and S3; the illustrative methodological sequence described in the main text is Table S4.

A note on the size of the evidence base. The extraction set comprises 79 records. Four of these were subsequently identified as duplicate entries for the same publication, so Appendix A of the main text lists 75 unique studies. The index values reported here were computed on the full 79-record set, and Section 3.7 of the main text states both figures so that the difference between the number of records analyzed and the number of publications listed is not read as an inconsistency.

S1.1 Translational Readiness Index (TRI)

TRI expresses the analogical readiness of each mechanism on a 0–100 scale. Five evidence dimensions were scored 0 = none, 1 = weak, 2 = moderate, 3 = strong from the extracted evidence and cited syntheses: D1 animal model, D2 human bereavement, D3 human–animal bond, D4 effect-size quantified, and D5 direct pet-loss evidence. Dimension scores were combined with weights emphasizing human-bereavement support (0.30), human–animal-bond support (0.25), and quantified effect size (0.30), with the remainder (0.15) on animal-model support. D5 (direct pet-loss evidence) equals 0 for every mechanism by construction, because no primary study has yet measured any of these markers in bereaved pet owners; the index therefore ranks analogical readiness for a first study, not demonstrated effects.

S1.2 First-Study Priority Index (FSPI)

FSPI = Readiness (TRI, rescaled 0–1 within the index) × Citation-consensus × Sample-adequacy. The three inputs are drawn from methodologically independent sources, so their product is informative rather than redundant:

- Readiness — the TRI above (literature synthesis).
- Citation-consensus — supporting / (supporting + contrasting) Smart Citations (Scite), aggregated across each mechanism's key papers (live citation-classification database).
- Sample-adequacy — min(1, median empirical N / 30) for the primary studies of the mechanism; reviews without a primary N are scored at a neutral 0.70.

Table S1. Base-scheme inputs and FSPI ranking (79-record extraction set).

Mechanism	Readiness (TRI)	Citation consensus	Median N	Sample adequacy	FSPI	Readiness rank	FSPI rank
HPA + autonomic	85	1.00	34	1.00	85.0	2	1
Neuroinflammatory	92	0.71	99	1.00	65.1	1	2
Oxytocinergic	70	0.81	30	1.00	56.6	3	3

Dopaminergic	58	0.75	24.5	0.82	35.7	4	4
Serotonergic	58	0.86	—	0.70	35.0	4	5
Endocannabinoid	43	1.00	—	0.70	30.1	6	6

FSPI = TRI × consensus × sample-adequacy (e.g., HPA 85 × 1.00 × 1.00 = 85.0; neuroinflammatory 92 × 0.71 × 1.00 = 65.1). Incorporating reliability and statistical power moves the HPA axis from readiness-rank 2 to FSPI-rank 1, while the neuroinflammatory system falls from 1 to 2.

S1.3 Sensitivity to weighting and to evidence-base size

Weighting sensitivity. The FSPI-rank of the HPA axis was recomputed under five alternative weighting schemes (equal weights; readiness-emphasized; consensus-emphasized; power-emphasized; and a consensus × power scheme with readiness down-weighted). The HPA axis ranked first in four of the five schemes, indicating the result is not an artifact of one weighting choice.

Stability across expansion. The ranking was recomputed as the evidence base grew (33 → 46 → 58 → 79 records). The HPA axis remained first throughout, and its lead over the neuroinflammatory system widened from 16 to 20 points as the neuroinflammatory literature accrued contrasting citations while the HPA literature did not; the reordering therefore sharpens rather than dissolves as evidence accumulates.

S1.4 Independent robustness analyses

Two analyses that use different inputs from the FSPI converged on the same conclusion. An evidence-triangulation analysis counted the distinct study designs supporting each mechanism (causal animal-loss model, human longitudinal, human experimental, cross-sectional, and synthesis review) plus a bonus for possessing a causal animal-loss anchor. A concentration analysis computed the Herfindahl index (HHI) of citation weight within each mechanism, quantifying how much of the evidence rests on a single dominant paper.

Table S2. Triangulation breadth and concentration risk.

Mechanism	Distinct designs	Causal animal-loss anchor	Triangulation score	Top-paper share	HHI (concentration)
HPA + autonomic	4	Yes	5	25%	0.19
Oxytocinergic	3	Yes	4	23%	0.16
Endocannabinoid	3	Yes	4	45%	0.29
Serotonergic	2	Yes	3	53%	0.36
Dopaminergic	2	No	2	55%	0.42
Neuroinflammatory	2	No	2	74%	0.57

The HPA axis is the only mechanism spanning four independent designs including a causal animal-loss model (Girard-Buttoz et al. 2021, early maternal loss and diurnal cortisol in wild chimpanzees), and is among the least concentrated. The neuroinflammatory system, first on naive readiness, is the most fragile under both lenses: it has no causal animal-loss anchor and 74% of its citation weight rests on one general, non-bereavement review of relationships and inflammation (Kiecolt-Glaser et al. 2010). FSPI, triangulation breadth, and low concentration are methodologically independent yet all favor the HPA axis; the three-way convergence, not any single index, is the basis for the recommendation.

Supplementary Figures

The two robustness analyses summarized in Section S1.4 are shown graphically below. Figure S1 gives the evidence-triangulation matrix underlying Table S2, and Figure S2 shows the mechanism co-occurrence network whose hub structure motivates measuring the HPA and oxytocinergic systems jointly.

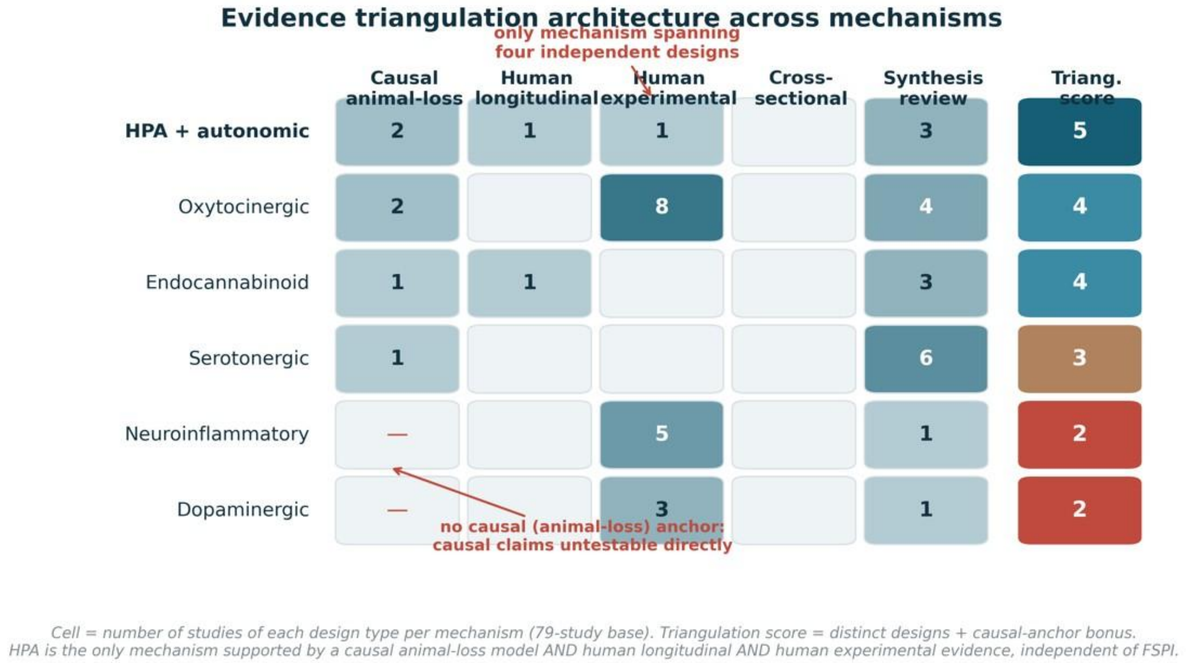

Figure S1. Evidence triangulation architecture across mechanisms. Each cell gives the number of studies of a given design type contributing to each mechanism within the evidence base, and the triangulation score is the count of distinct design types plus a bonus for a causal animal-loss anchor. The HPA axis is the only mechanism supported by a causal animal-loss model together with human longitudinal and human experimental evidence, a consilience that is independent of the First-Study Priority Index. The neuroinflammatory and dopaminergic mechanisms lack any causal animal-loss anchor, so their causal claims cannot be tested directly in a loss model.

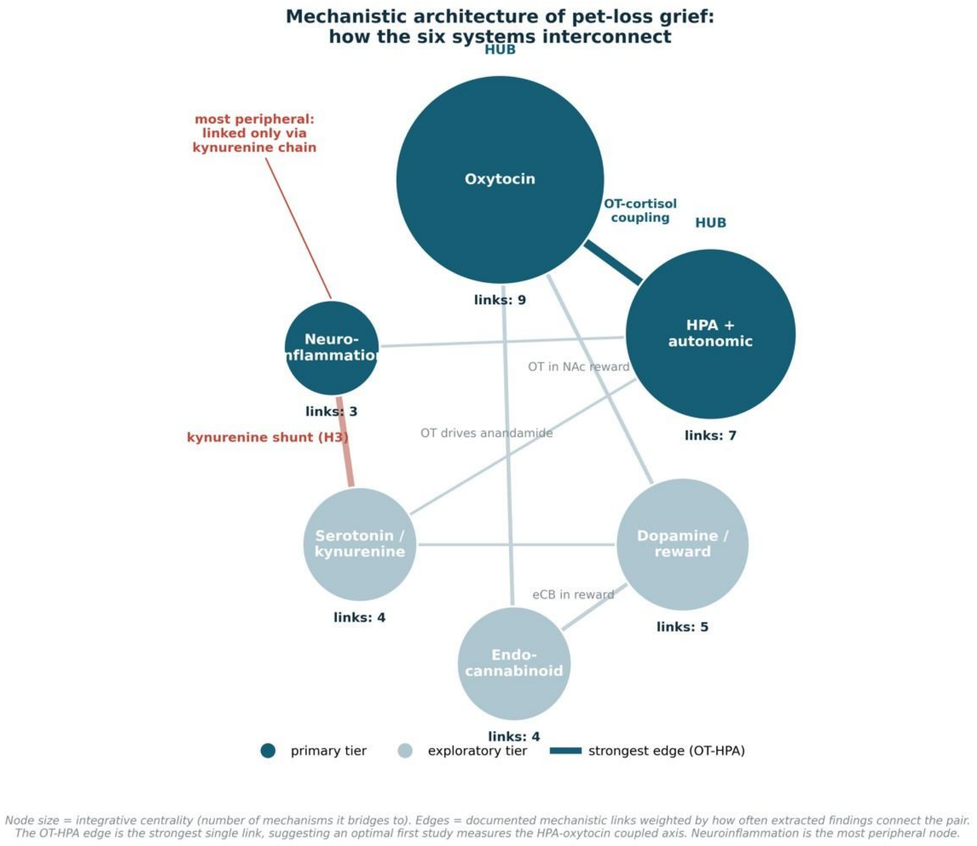

Figure S2. Mechanistic architecture of pet-loss grief and how the six neurobiological systems interconnect. Node size denotes integrative centrality, the number of other mechanisms to which a system is documented to bridge, and edges denote mechanistic links weighted by how often the extracted findings connect the pair. Oxytocin and the HPA axis are the two hub mechanisms, and the oxytocin-cortisol coupling is the single strongest edge, which motivates measuring the HPA-oxytocin coupled axis jointly rather than the HPA axis in isolation. Neuroinflammation is the most peripheral node, connected only through the kynurenine chain to serotonin.

S1.5 Figure-value provenance

Every quantitative value shown in Figures 1–6 is either (i) an estimate extracted directly from a cited primary study or synthesis, or (ii) a transparent calculation from such values. No figure value is invented. Interpolated or indicative quantities are flagged explicitly, both here and in the main text. Table S3 is a master index; the notes that follow give the derivation and citation for each figure.

Table S3. Master provenance index for Figures 1–6.

Fig.	Value(s)	Source (author–year)	Derivation	Manuscript location
1	32.6% experienced pet death; 7.5% prolonged grief; RR 1.27; 8.1% population-attributable; N = 975	Hyland 2026	Single-source estimates read directly from one UK quota-panel study	§1, §4.2 / legend
2	TRI, consensus, median N, FSPI per system	Table S1 (this doc)	Composite heuristics; see S1.1–S1.2	§3.7 / legend
3	0.76; 0.94; 0.72 chain confidence	Dantzer 2011; Cervenka 2017	0.76 = 16/(16+5); 0.94 = 17/(17+1); 0.72 = 0.76 × 0.94	§3.5, §3.7 / legend
4	FSPI bars 85/65/57/36/35/30; bubble x/y/size	Table S1	TRI × consensus × sample-adequacy	§3.7 / legend
5	Top-paper share 74–23%; HHI 0.57–0.16; rank matrix	Table S2	Concentration = HHI of citation weight; ranks from three analyses	§3.7 / legend
6	7.5% / 9.4% pet; 9.8% CG; 3.6% DSM-5; 29.3% COVID	Hyland 2026; Lee 2020; Lundorff 2017; APA 2013; Tang & Xiang 2021	Reported point estimates; DSM-5 value is a range midpoint	§4.2 / legend

Figure 1 — One Health framework

All prevalence statistics in the stat band are single-source, from one large UK quota-panel study (Hyland 2026, N = 975): 32.6% of respondents had experienced the death of a pet; conditional prolonged-grief rate 7.5%; relative risk of prolonged grief disorder after pet loss versus non-bereaved 1.27; and a population-attributable share of 8.1% of all prolonged-grief cases. Because these values share one source, the legend names that source and sample rather than implying meta-analytic support. The five shared-substrate icons (oxytocinergic, HPA-axis, endocannabinoid, dopaminergic, inflammatory) are the qualitative organizing scheme of the review, not quantities.

Figure 2 — Mechanism map

Each system's four data columns (readiness TRI, citation consensus, median N, FSPI) are reproduced verbatim from Table S1. Exact values: HPA 85 / 1.00 / 34 / 85.0; neuroinflammatory 92 / 0.71 / 99 / 65.1; oxytocinergic 70 / 0.81 / 30 / 56.6; dopaminergic 58 / 0.75 / 24.5 / 35.7; serotonergic 58 / 0.86 / — / 35.0; endocannabinoid 43 / 1.00 / — / 30.1. The primary/exploratory tier split follows the two-tier framing introduced in Section 3. Median N is shown as an em dash where a mechanism rests on review-level evidence without an extractable primary sample.

Figure 3 — Kynurenine pathway (serial-chain confidence)

The pathway steps, namely pro-inflammatory induction of indoleamine 2,3-dioxygenase (IDO), the tryptophan-to-kynurenine shunt, accumulation of neurotoxic quinolinic acid, and the resulting serotonergic deficit, are established mechanisms reviewed in Dantzer 2011, Myint & Kim 2014, and Cervenka 2017. The two link-level numbers are citation-consensus ratios (supporting / [supporting + contrasting] Smart Citations): the inflammation→kynurenine link is anchored on Dantzer 2011 with 16 supporting and 5 contrasting citations, $16/(16+5) = 0.762 \approx 0.76$; the kynurenine→serotonin link is anchored on Cervenka 2017 with 17 supporting and 1 contrasting, $17/(17+1) = 0.944 \approx 0.94$. Because the hypothesis is that these links operate in series, the two ratios multiply: $0.76 \times 0.94 = 0.716 \approx 0.72$. Serial hypotheses cap low by construction, since any additional unmeasured link would multiply confidence down further. That the full cascade is established in the general stress-immunology literature but has not been measured in bereaved pet owners is stated in Section 3.5 of the main text. Following review, the figure legends were restricted to description alone, and interpretive statements were relocated to the main text.

Figure 4 — FSPI prioritization

Bubble position encodes analogical readiness (x-axis, TRI) and citation consensus (y-axis); bubble area encodes sample-adequacy. The bars are the composite FSPI from Table S1 (HPA 85.0, neuroinflammatory 65.1, oxytocinergic 56.6, dopaminergic 35.7, serotonergic 35.0, endocannabinoid 30.1), each shown with its literal computation (e.g., HPA "85 × 1.00 × 1.00"). The result that the HPA axis is prioritized even though the neuroinflammatory system leads on readiness alone is the readiness-rank versus FSPI-rank reversal documented in Table S1 and Section S1.2, and it is stated in Section 3.7 of the main text. All values are reproducible from Section S1.2.

Figure 5 — Robustness (concentration and convergence)

The left panel plots top-paper share and the Herfindahl index (HHI) of within-mechanism citation weight from Table S2: neuroinflammatory 74% / 0.57 (dominant paper Kiecolt-Glaser et al. 2010, a general relationships-and-inflammation review, not bereavement-specific), dopaminergic 55% / 0.42 (Kakarala 2020), serotonergic 53% / 0.36 (Cervenka 2017), endocannabinoid 45% / 0.29 (Don 2015), HPA 25% / 0.19 (Seiler 2020 plus two primary bereavement-cortisol studies), oxytocinergic 23% / 0.16 (Handlin 2011 plus many). The right panel is a rank matrix across three methodologically independent analyses (FSPI, evidence-triangulation breadth, and citation concentration; 1 = best); the HPA axis ranks 1/1/2 and is the only mechanism placed in the top band by all three, which is the convergence result.

Figure 6 — Prevalence forest

Point estimates are reported values from their respective studies: pet loss 7.5% (Hyland 2026, N = 975, conditional ICD-11 prolonged-grief rate) and 9.4% (Lee 2020, N = 438, DSM-5 persistent complex bereavement items); human complicated grief 9.8% (Lundorff et al. 2017, meta-analytic pooled prevalence); and COVID-19 bereavement 29.3% (Tang & Xiang 2021, N = 422, Traumatic Grief Inventory–Self Report). Two honesty flags apply and are stated in Section 4.2 of the main text: (i) the DSM-5 point of 3.6% is the midpoint of the 2.4–4.8% persistent-complex-bereavement reference range (APA 2013), not a study estimate; and (ii) the human-benchmark intervals are reported ranges or indicative confidence intervals rather than uniformly re-derived 95% CIs. The pet-loss interval shown for Hyland 2026 is an indicative binomial (Wilson) interval around the 7.5% conditional rate at N = 975, since the source reports a conditional rate; it should be read as approximate.

Attribution note. The COVID-19 point was labeled "Chen et al. 2021" in an earlier figure draft. The 29.3% (TGI-SR) / N = 422 estimate is from Tang & Xiang (2021), Globalization and Health; "Chen" appears only as a co-author on a companion report from the same cohort. Figure 6 was corrected accordingly and now reads "Tang & Xiang 2021".

S1.6 Stated limitations

- (i) TRI and FSPI are proposed heuristics, not established metrics, and the dimension weights are the authors' choice (mitigated by the five-scheme sensitivity analysis in S1.3).
- (ii) Every input is built on human-bereavement and human–animal-bond data; direct pet-loss biomarker studies do not exist, so the indices indicate where a first study should begin, not that any mechanism has been demonstrated in pet loss.
- (iii) The cross-mechanism ranking is bounded by n = 6 mechanisms; additional studies stabilize the within-mechanism consensus and sample estimates but cannot change the six-point ordering.
- (iv) Citation consensus was found to be orthogonal to both citation volume and sample size (both relationships statistically indistinguishable from zero), so neither popularity nor sample size substitutes for direct evidence of agreement. Consensus values are computed from automated Smart Citation classifications (Scite), which carry a nonzero misclassification rate; they are therefore treated as an approximate signal, and the recommendation rests on the agreement of three independent analyses rather than on any single consensus value.
- (v) Four records in the extraction set were duplicate entries for the same publication. They were retained in the computation of the indices reported here and consolidated in Appendix A of the main text, which lists 75 unique studies. The effect of consolidation on the index values has not been re-tested.

S1.7 References cited in this supplement

American Psychiatric Association. Diagnostic and Statistical Manual of Mental Disorders, 5th ed. (DSM-5). Arlington, VA: American Psychiatric Publishing; 2013.

Cervenka I, Agudelo LZ, Ruas JL. Kynurenines: Tryptophan's metabolites in exercise, inflammation, and mental health. Science. 2017;357(6349):eaaf9794. doi:10.1126/science.aaf9794.

Dantzer R, O'Connor JC, Lawson MA, Kelley KW. Inflammation-associated depression: from serotonin to kynurenine. Psychoneuroendocrinology. 2011;36(3):426–436. doi:10.1016/j.psyneuen.2010.09.012.

Girard-Buttoz C, Tkaczynski PJ, Samuni L, et al. Early maternal loss leads to short- but not long-term effects on diurnal cortisol slopes in wild chimpanzees. eLife. 2021;10:e64134. doi:10.7554/eLife.64134.

Hyland P. No pets allowed: Evidence that prolonged grief disorder can occur following the death of a pet. PLoS ONE. 2026;21:e0339213. doi:10.1371/journal.pone.0339213.

Kiecolt-Glaser JK, Gouin JP, Hantsoo L. Close relationships, inflammation, and health. Neurosci Biobehav Rev. 2010;35(1):33–38. doi:10.1016/j.neubiorev.2009.09.003.

Lee SA. Does the DSM-5 grief disorder apply to owners of deceased pets? A psychometric study of impairment during pet loss. Psychiatry Res. 2020;285:112800. doi:10.1016/j.psychres.2020.112800.

Lundorff M, Holmgren H, Zachariae R, Farver-Vestergaard I, O'Connor M. Prevalence of prolonged grief disorder in adult bereavement: A systematic review and meta-analysis. J Affect Disord. 2017;212:138–149. doi:10.1016/j.jad.2017.01.030.

Myint AM, Kim YK. Network beyond IDO in psychiatric disorders: Revisiting neurodegeneration hypothesis. Prog Neuropsychopharmacol Biol Psychiatry. 2014;48:304–313. doi:10.1016/j.pnpbp.2013.08.008.

Tang S, Xiang Z. Who suffered most after deaths due to COVID-19? Prevalence and correlates of prolonged grief disorder in COVID-19 related bereaved adults. Global Health. 2021;17:19. doi:10.1186/s12992-021-00669-5.

Additional papers named in the notes above (Handlin 2011, Seiler 2020, Kakarala 2020, Don 2015, Fagundes et al. 2013, Nagasawa et al. 2015, Border et al. 2019) are cited in full in the main manuscript reference list. Consensus and concentration statistics were extracted from Scite Smart Citations during manuscript preparation.

Table S4. Illustrative methodological sequence for culturally adapted instrument development

Table S4. Illustrative methodological sequence for culturally adapted pet loss grief assessment in Korean and East Asian populations. The sequence reflects methodological dependencies, and the activities and outputs are examples rather than a prescribed research timetable.

Methodological step	Dependency	Illustrative activities and outputs
Conceptual exploration	Initial step	Focus groups and interviews with bereaved Korean pet owners to identify locally relevant grief expressions and cultural barriers. Potential output: conceptual model and preliminary item pool
Translation and cultural adaptation	After conceptual exploration	Forward-backward translation and cultural adaptation of PBQ, CABS, and ICG, supported by cognitive interviews with bilingual participants. Potential output: Korean-language candidate measures
Psychometric evaluation	After adaptation	Pilot testing, confirmatory factor analysis, reliability assessment, and convergent and discriminant validity testing. Potential output: validated Korean instruments
Normative evaluation	After validation	Population-based survey to establish Korean norms and preliminary cutoff scores. Potential output: Korean normative data
Cross-cultural extension	After Korean validation	Collaboration with Japanese, Chinese, and Taiwanese researchers to assess measurement equivalence. Potential output: evidence supporting regional adaptation

Section 5.2 of the main text refers to this table as an illustration of the methodological steps that may support culturally adapted assessment.
